# Further Validation of a Rapid Screening Semiquantitative Thin-Layer Chromatographic Method for Marketed Antimalarial Medicines for Adoption in Malawi

**DOI:** 10.1155/2018/2130390

**Published:** 2018-05-02

**Authors:** Dorcas Osei-Safo, Ibrahim Chikowe, Jerry Joe Ebow Kingsley Harrison, Daniel Konadu Yeboah, Ivan Addae-Mensah

**Affiliations:** ^1^Chemistry Department, School of Mathematical and Physical Sciences, College of Basic and Applied Sciences, University of Ghana, Accra, Ghana; ^2^Biomedical Sciences and Health Professions, College of Medicine, University of Malawi, Blantyre, Malawi

## Abstract

A recently developed semiquantitative thin-layer chromatographic (SQ-TLC) assay has been employed in postmarketing surveillance of antimalarial medicines used in Malawi prior to HPLC assay. Both methods gave analogous results in a significant majority of the samples, with a good correlation (*r* = 0.9012) for the active pharmaceutical ingredients of the dosage forms assayed. Artemether-containing medicines had the highest percentage (92.67%) of samples with comparable results for both assays. The lowest percentage (66.67%) was observed in artesunate-containing medicines. The SQ-TLC method was validated for specificity, accuracy, precision, linearity, and stability according to the International Conference on Harmonisation guidelines, with the results falling within acceptable limits. For specificity, retention factor values of the test samples and reference standards were comparable, while accuracy and precision of 91.1 ± 5.7% were obtained for all samples. The method was linear in the range 1.0–2.0 *µ*g/spot with a correlation coefficient of *r* = 0.9783. Stability tests also fell within acceptable limits. In this study, we present the validation of the SQ-TLC method and propose its adoption as a rapid screening tool for field estimation of the quality of antimalarial and other essential medicines in Malawi and other parts of the developing world prior to a more accurate HPLC assay.

## 1. Introduction

Developing countries continue to be a suitable hub for pharmaceutical counterfeiting due to poor policing and postmarketing surveillance of pharmaceuticals. This practice jeopardises public health efforts in ameliorating disease burden on populations. With the rate of resistance to essential medicines being far higher than the rate of development of new drugs, it is important that techniques that allow regular and effective surveillance of medicines on the market are constantly developed and updated. Also, new strategies to ensure patient compliance should be devised to prolong the lifespan of these medicines before they are lost ultimately to resistance. Thin-layer chromatography (TLC) finds wide application in the analysis of active pharmaceutical ingredients (APIs) and pharmaceutical dosage forms. Regardless of the superiority of high-performance liquid chromatography (HPLC), TLC continues to be a mandatory identification and purity test in pharmaceutical analysis. It has been successfully employed as a simple, robust, and cheap method for providing product quality assessments including the identification of substances resulting from degradation as well as the detection of counterfeit and subtherapeutic concentrations of essential medicines [1–5].

Many of the interventions proposed and deployed to mitigate this problem through the support of the World Health Organization (WHO) and other international organisations in resource-constrained settings are TLC-based. One such prominent initiative is the Global Pharma Health Fund (GPHF) Minilab which contains consistent, cheap, and simple methods for the prompt detection of counterfeit and substandard medicines for tuberculosis, malaria, and HIV/AIDS, antibiotics, and other essential medicines. It is important to note that the minilab protocols are updated regularly to address current needs [6]. Many other laboratories have also developed various effective qualitative and SQ-TLC methods aimed at rapid screening of pharmaceuticals in low income-earning countries [3, 4]. The increased level of reporting of pharmaceutical crime must be matched with a continuous development of simpler, more reliable, and more affordable protocols for more effective medicine quality monitoring.

A recent postmarketing surveillance of antimalarial drugs distributed in Malawi employed a computer-based TLC spot fading technique in an SQ-TLC determination of the API [7]. The SQ-TLC results obtained were then confirmed by HPLC methods and further validated according to the International Conference on Harmonisation (ICH) guidelines. The findings from the study suggested the existence of substandard antimalarial medicines attributed to the presence of both excessive and insufficient API. The country was also found to be heavily reliant on imported antimalarial medicines, a situation which demands an urgent need to carry out regular and thorough postmarket surveillance of medicines to ensure better quality medicines. Being a developing country, Malawi faces the usual challenges regarding a proper regulatory framework for medicines.

As part of efforts to boost the regulatory activities to the required scale and frequency, we present the SQ-TLC method and recommend its adoption in both field and laboratory evaluation of the quality of essential medicines used in Malawi. The method is useful for rapid and reliable estimation of the quality of antimalarial drugs prior to a more accurate HPLC assay and has been successfully employed in two surveys in Ghana and Togo sponsored by the WHO and the West African Health Organization (WAHO) [8, 9].

## 2. Materials and Methods

### 2.1. Antimalarial Medicines

One hundred and twelve antimalarial medicines, comprising both artemisinin-based and non-artemisinin-based samples, were analysed (Table 1). They were purchased from licensed and unlicensed markets from the National Malaria Control Programme strategy-partitioned zones designated as southwest, southeast, central, and north zones of the country [7].

### 2.2. Reference Standards

The reference standard (RS) for each sample analysed was obtained from the WHO International Chemical Reference Substance (ICRS) of the European Directorate for the Quality of Medicines & HealthCare (EDQM), Strasbourg, France.

### 2.3. Semiquantitative Thin-Layer Chromatography (SQ-TLC) Assay

#### 2.3.1. Materials

The assay was carried out at room temperature in TLC glass tanks (22.5 × 10 cm) lined with a filter paper and saturated with the mobile phase. Two proposed solvent systems were selected as the mobile phase for each sample API, and the recommended visualising agents were employed (Table 2) [10]. Silica gel 60 F_254_ (Merck) or silica gel 60 (Fluka) precoated on the aluminium foil served as the stationary phase.

#### 2.3.2. Preparation of Dosage Form and Reference Standard Solutions

An amount of the powdered dosage form equivalent to 10 mg of a given API was weighed into a clean dry beaker and dissolved in 5 mL of a suitable solvent. The mixture was shaken for at least 5 minutes and filtered. Another 3 mL of the solvent was added to the residue in a beaker, shaken gently, allowed to settle for at least 5 minutes, and filtered. To check the effectiveness of the extraction, another 2 mL of the solvent was added to the residue and a TLC spot developed from the subsequent filtrate alongside the reference standard. The absence of a spot from this filtrate suggested complete API extraction. The various filtrates were then added together and made up to 10 mL to give the dosage form solution. So, in effect, a 1 mg/mL dosage form solution was prepared. For a fixed dose combination formulation, its component APIs were assayed independently of the each other. For example, if an artemether/lumefantrine-containing sample was assayed for artemether, an amount of the powdered dosage form equivalent to 10 mg of artemether API was weighed, regardless of how much of lumefantrine would be present in the weighed amount. A suitable solvent (in this case, ethyl acetate) was then added to exhaustively extract the artemether API to obtain the 1 mg/mL test solution. Likewise, when lumefantrine was assayed, an amount of the powdered dosage form equivalent to 10 mg of lumefantrine API was weighed and taken through a similar process to obtain the 1 mg/mL test solution. Solutions (1 mg/mL) of reference standards of the APIs of each sample were also prepared and stored at 4°C.

#### 2.3.3. Spotting, Development, and Visualisation of TLC Plates

While maintaining a fixed volume (2 *µ*L) of the test solution, increasing volumes of the reference standard, ranging from 1.0 to 2.4 *µ*L of the 1 mg/mL solutions, were spotted onto the plate using a Hamilton microsyringe. Thus, the volume in *µ*L corresponded to the weight of substance in *µ*g. The recommended experimental conditions in Table 2 were employed in the development and visualisation of the TLC plates.

#### 2.3.4. SQ-TLC Estimation

**Figure 1:**
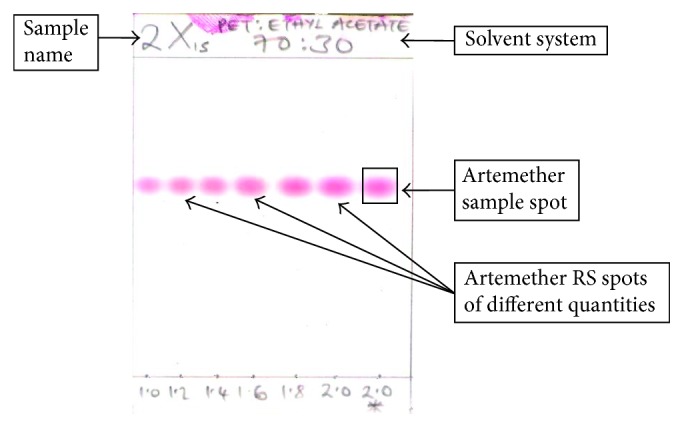
A sample of a developed TLC plate of an artemether-containing drug.

The chromatograms were immediately scanned and saved on a computer. Using Microsoft Office Picture Manager, the brightness of the spots on the developed chromatograms was varied gradually, and the rates at which the reference standard and test solution spots faded were compared simultaneously. The principle of the method is that the reference standard and the test solution spots on the chromatogram that had the same concentration would fade and finally disappear at the same time. For example, if a spot of a test solution is presumed to contain 2 *µ*g, it must fade and eventually become invisible at the same time as 2 *µ*g of the reference standard. A sample of a developed TLC plate of an artemether-containing antimalarial drug can be found in Figure 1.

The concentration of a test solution is estimated by taking note of the reference standard spot that begins to fade simultaneously with the spot of the test solution and reference standard spot that fades completely after the spot of the test solution. This gives the range in which the amount of the API in the test solution falls. For example, if the spot of the test solution (2 *µ*L) of a sample containing 200 mg of an API is expected to fade at the same time as the 2 *µ*L reference standard spot, which fades between 1.4 *µ*L and 1.6 *µ*L spots of the reference standard, then

  1.4 *µ*L = lower limit;
  1.6 *µ*L = upper limit of the API content of the test solution.

In percentage equivalent,

  lower limit = 1.4/2.0 × 100 = 70.0%;
  upper limit = 1.6/2.0 × 100 = 80.0%.

Converting percentage to mass,

  lower limit = 1.4/2.0 × 200 mg = 140 mg;
  upper limit = 1.6/2.0 × 200 mg = 160 mg.

This implies that the percentage of API in the test solution is between 70 and 80% of the manufacturer's label claim. Hence, the actual amount of API in the tablet claimed by the manufacturer to contain 200 mg is between 140 and 160 mg. Such a sample will be deemed to be noncompliant with The International Pharmacopoeia requirement that stipulates that each tablet must contain not less than 90% and not more than 110% of the manufacturer's label claim. Each test sample analysis was replicated 12 times, 6 from each of the two solvent systems presented in Table 2. The average for the upper and lower limits was then calculated for each sample to obtain the average API quantity.

#### 2.3.5. SQ-TLC Method Validation

Validation for the SQ-TLC method was performed according to the International Conference on Harmonisation (ICH) guidelines, and the validation parameters considered were specificity, accuracy, precision, linearity, and stability [2, 11]. Specificity was determined by comparing the retention factor (Rf) values of the test sample and reference standard prepared under the same conditions (concentration, dissolution solvent, volume, temperature, and time), as shown in the sample chromatogram presented in Figure 1. The measurement of accuracy was performed by comparing the SQ-TLC results to those of the gold standard method of HPLC. In addition, a reference standard was run simultaneously alongside the sample analyte for comparison. Precision was found by calculating the average relative standard deviation (RSD) values for each solvent system (*n* = 6), giving a total of 12. The concentration of the TLC spots were found to be proportional to their brightness such that a linear relationship was obtained in the range 1.0–2.0 *µ*g/spot. The stability of the analyte in both the test sample and the reference standard was checked through retesting of every 5th sample after 2 hours, 4 hours, and 24 hours, while chromatograms were evaluated after 30 minutes, 2 hours, and 4 hours after final drying.

### 2.4. HPLC Assay

In order to authenticate the results of the SQ-TLC assay, the sample API contents were assayed using HPLC procedures suitable for each particular API, adopted from the pharmacopoeias and literature [7]. In the HPLC analysis, each API had 6 replicates, and the mean and standard deviation were computed.

### 2.5. Data Interpretation

The component API of a dosage form was classified as “compliant (C)” if its quantity fell within acceptable limits of the Ph. Int. (90–110%); “noncompliant (NC)” if the quantity was greater than (overdose) or less than (underdose) the acceptable limits; and “borderline compliant (BLC)” if the amount was marginally compliant with Ph. Int. limits by **±5%** for SQ-TLC and **±2%** HPLC. For the combination therapies, a sample was considered as “compliant” if all component APIs were compliant; “NC” if ≥1 of the API components was “NC”; and “BLC” if all components were separately “BLC” or ≥1 is “BLC” amongst “C” components. The data for SQ-TLC and HPLC were further analysed for correlation. All the data were analysed using Microsoft Excel 2007.

## 3. Results and Discussion

### 3.1. Sample Composition

The 112 antimalarial samples were either monotherapy (containing one API) or combination therapy (containing two or three APIs). The samples comprised tablets, suspensions, injections, and mixtures containing sulphadoxine, pyrimethamine, quinine, lumefantrine, piperaquine, sulphamethoxypyridazine, artemether, artesunate, and dihydroartemisinin APIs. Table 1 shows the sample composition.

A total of 101 out of 112 antimalarial samples consisting of 207 APIs were analysed by both methods (SQ-TLC and HPLC). Sulphamethoxypyridazine-containing samples (5) gave a spot with the same retention time as sulphadoxine-containing samples on TLC but failed to stain well with I_2_/KI to enable an effective semiquantitative analysis. Quinine sulphate samples (6) could also not be analysed by SQ-TLC due to technical reasons. In both cases, however, HPLC analysis was successful. The piperaquine reference substance was unavailable; therefore, dihydroartemisinin/piperaquine samples were evaluated based on the quality and quantity of only the dihydroartemisinin component.

### 3.2. Summary of Validation Parameters Tested for SQ-TLC Assay

The SQ-TLC method, in terms of its specificity, accuracy, precision, linearity, and stability, met the ICH requirements. Rf values of the test samples and their corresponding reference standards were very similar as shown in Figure 1, supporting specificity. The method enabled the successful assay of the test samples with good accuracy and precision of 91.1 ± 5.7% for 12 determinations of all 207 APIs. The amount of API in a TLC spot was found to be linear with its brightness in the range 1.0–2.0 *µ*g/spot with a good correlation coefficient of *r* = 0.9783. Stability test results fell within acceptable limits. The analytes were stable for at least 5 hours during sample preparation, 2 hours on the sorbent surface before development, at least 5 hours during development, and at least 2 hours on the plate after chromatography. Moreover, there was no single sample with degradation products seen on any of the chromatograms.

### 3.3. Comparison of SQ-TLC and HPLC Results

Each of the 207 APIs of the various pharmaceutical dosage forms was assayed by SQ-TLC, and the results were validated by HPLC.  Table 3 is a representation of the results obtained. For combination therapies, the API being assayed is in bold. The upper section of the table shows samples with similar API quantities from both assays. For example, the sample 2_3_X_1_ (ATM/LUM: 20/120 mg) was found to contain 103.0% of lumefantrine by SQ-TLC. The HPLC analysis of the same sample also gave 103.3% of lumefantrine. The International Pharmacopoeia requirement for lumefantrine tablets is at least 90% and at most 110% of the manufacturer's label claim of lumefantrine API in a given dosage form.

Therefore, both the SQ-TLC and HPLC assays showed that the sample 2_3_X_1_ contained the requisite amount of lumefantrine and was therefore found to be compliant The reason for setting a wider margin for the SQ-TLC technique was to take care of the inherent subjectivity of the technique. Spotting of the plates with microsyringes, application of visualising agents, heating of plates in the oven, and fading of the spots using Microsoft Office Picture Manager were likely avenues for the introduction of errors in the method as compared to the HPLC method. The SQ-TLC method relies on limits determined by specific amounts of RS for which only certain realistic quantities are possible due to precision. Thus, some samples which were borderline compliant by SQ-TLC turned out to be either noncompliant or compliant by HPLC. The pyrimethamine component in the sample 4_3_Y_12_ (ATS/SM/PYR: 100/250/12.5 mg) was borderline compliant by SQ-TLC (115%) but was noncompliant by HPLC (113%), while the same API in the sample 3_2_P_10_ (SDX/PYR: 500/25 mg), found to be borderline compliant by SQ-TLC (89.5%), was compliant by HPLC (94%). Similarly, some SQ-TLC compliant samples were either borderline compliant or noncompliant by HPLC and vice versa. Therefore, the SQ-TLC method would be most suitable for detecting grossly substandard medicines—either heavily underdosed or overdosed—while samples whose API content falls within an estimation of 80–120% should be further analysed by HPLC to confirm the results. The method can also be used for preliminary screening on the field by medicine quality assurance inspectors for preliminary decision-making, prior to further HPLC instrumental analysis, or be used for reasonable decision-making in circumstances of nonavailability of more sophisticated methods such as HPLC.

The assay results for all the 207 APIs are presented in Supplementary 1. Artemether-containing dosage forms gave the highest percentage (92.67%) of samples whose API content fell within the same quality category. Out of this number, 31.70% of the API were complaint, while 60.97% were noncompliant in both assays. Artesunate-containing samples gave the lowest percentage (66.67%) of samples with comparable results.

**Figure 2:**
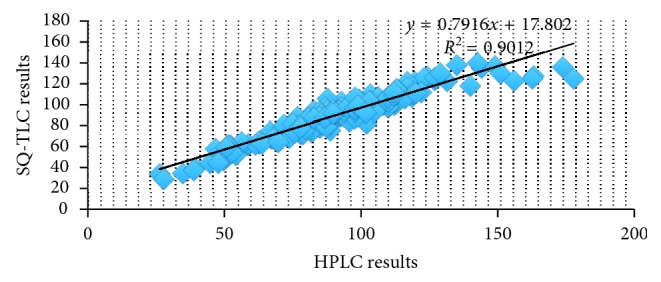
Correlation between HPLC and SQ-TLC results for API components of antimalarial medicines.

A comparison of data from the two assays showed that generally, the results of the SQ-TLC assay were largely confirmed by the HPLC results.  Figure 2 shows that there was a good correlation (*r* = 0.9012) between API quantities in the dosage forms assayed by both methods.

**Figure 3:**
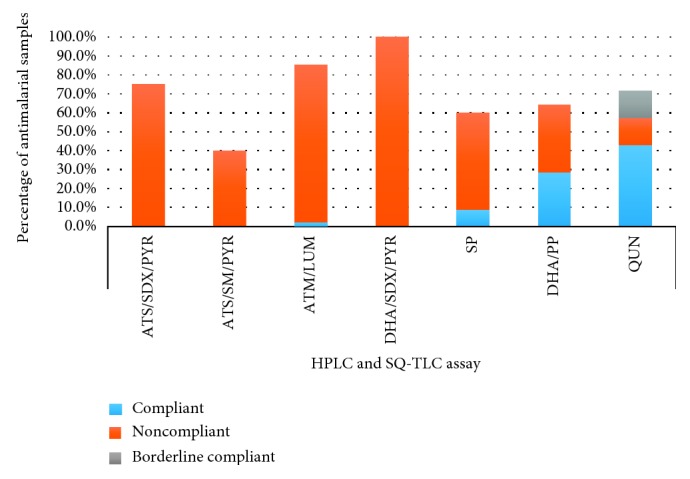
Antimalarial medicines with same results from SQ-TLC and HPLC assays.

Since the therapeutic effect of a combination therapy is influenced by the quality of its individual API components, the antimalarial samples were further analysed based on the contribution from their component APIs. Any sample that was identified with one or more noncompliant API was automatically considered noncompliant.  Table 4 shows conclusions drawn on the quality of some combination therapies (see Supplementary 2), while Figure 3 depicts a graphical presentation for all the antimalarial samples.

From Figure 3, all dihydroartemisinin/sulphadoxine-pyrimethamine samples (100%) gave the same results. However, the pyrimethamine component was compliant in all the 12 samples by both SQ-TLC and HPLC, and the dihydroartemisinin component was equally noncompliant, resulting in total failure.

Percentages of the same samples of the remaining antimalarial medicines that gave same conclusions from both assays were as follows: artemether/lumefantrine (85.4%, 35/41), artesunate/sulphadoxine-pyrimethamine (75.0%, 3/4), quinine (71.4%, 5/7), dihydroartemisinin/piperaquine (64.3%, 9/14), sulphadoxine-pyrimethamine (60.9%, 14/23), and artesunate/sulphamethoxypyrazine/pyrimethamine (40.0%, 2/5). Dihydroartemisinin/piperaquine samples were analysed based on only the dihydroartemisinin API due to the absence of a PPQ reference standard. Similarly, the sulphamethoxypyrazine component of the artesunate/sulphamethoxypyrazine/pyrimethamine sample failed to stain well in KI/I_2_ to enable a good estimation on TLC and so was excluded in the analysis. Conclusions were drawn based on the other two components.

### 3.4. Cost-Benefit Analysis of the SQ-TLC Method

TLC methods are generally simpler and cheaper than HPLC, requiring less expertise and instrumentation. The running costs involve TLC plates, low-grade solvents, and visualising agents which are much less expensive than the columns and specific-grade solvents for HPLC analyses. The anticipated limitations of accurately spotting the plate and subjectivity in the visualisation of the spots can be minimised with little practice. An estimated comparison of the cost drivers for HPLC and SQ-TLC analyses is presented in Table 5. Some common tools for the methods such as syringes and reference standards are excluded.

## 4. Conclusion

The results of the SQ-TLC estimation of quantities of active pharmaceutical ingredients in the antimalarial dosage forms analysed in this study were largely confirmed by HPLC quantification. The study has further validated the SQ-TLC technique as a rapid screening tool that can be adopted for use in resource-constrained communities to reliably detect substandard antimalarial medicines. The study further shows that the individual components of multicomponent fixed-dose combination regimens can be determined independently of each other using different extraction techniques. Samples with doubtful results could then be confirmed with HPLC or other more accurate methods. It is believed that this would help cut down on cost and lead to increased frequency of application of quality assurance methods to our essential medicines. The alarming rate and frightening scale-up in illicit trade and distribution of poor-quality medicines demand a relentless effort to continue to develop simpler, quicker, cheaper, and more reliable techniques to fight the scourge. Malawi stands to benefit if this technique is adopted as part of its malaria-control strategies.

## Figures and Tables

**Table 1:** Composition of analysed antimalarial medicines.

Non-ACTs		Doses copacked on one blister		Fixed-dose ACTs	
API	Number	API	Number	API	Number
Quinine sulphate	6	Ats copacked with SP	4	Atm/Lum	41
Quinine hydrochloride	3			Dha/Pp	14
Quinine bisulphate	4			Dha/SP	12
SP	23			Ats/SmP	5
Total	**36**		**4**		**72**

Atm, artemether; Ats, artesunate; Lum, lumefantrine; Dha, dihydroartemisinin; S, sulphadoxine; P, pyrimethamine; Pp, piperaquine phosphate; Sm, sulphamethoxypyridazine.

**Table 2:** Solvent systems for antimalarial active pharmaceutical ingredients.

API	Solvent system 1 (S1)	Solvent system 2 (S2)	Spraying reagent	Colour
Artesunate	Ethanol:ammonia (100 : 0.5)	Ethanol:toluene:ammonia (70 : 30 : 1.5)	Anisaldehyde/methanol	Purple
Artemether	Petrol:ethyl acetate (70 : 30)	Toluene:ethyl acetate (70 : 30)	Anisaldehyde/methanol	Purple
Artenimol	Toluene:ethyl acetate (60 : 40)	Toluene:ethyl acetate (70 : 30)	Anisaldehyde/methanol	Purple
Quinine	Methanol:ammonia (100 : 1.5)	Ethyl acetate:acetic acid:water (60 : 20 : 20)	I_2_/KI	Brown
Pyrimethamine	Ethyl acetate:methanol:ammonia (80 : 15:5)	Ethyl acetate:acetic acid:water (60 : 20 : 20)	I_2_/KI	Brown
Sulphadoxine	Ethyl acetate:methanol:ammonia (80 : 15:5)	Ethyl acetate:methanol:acetic acid (75 : 25 : 1)	I_2_/KI	Brown
Lumefantrine	Ethyl acetate:acetic acid:toluene (4 : 2 : 18)	Ethyl acetate:acetic acid (10 : 5)	I_2_/KI	Brown

**Table 3:** Comparison of API content of dosage forms by SQ-TLC and HPLC methods.

Sample code	Manufacturer's label claim (mg)	SQ-TLC assay (*n* = 12), % composition	Remark	HPLC assay (*n* = 6), % composition	Remark
*Samples with same results from both assays*					

4_2_Y_12_	**ATS**/SM/PYR: **200**/500/25^∗^	91.0 ± 5.5	C	92.77 ± 0.02	C
1_12_X_1_	ATM/**LUM**: 80/**480**	102.0 ± 4.9	C	102.5 ± 0	C
1_13_X_1_	ATM/**LUM**: 80/**480**	97.5 ± 5.1	C	109.96 ± 0.0	C
2_3_X_1_	ATM/**LUM**: 20/**120**	103.0 ± 4.4	C	103.3 ± 0.3	C
1_6_Z_1_	**DHA**/PPQ: **40**/320	97.0 ± 7.7	C	97.5 ± 0.9	C
1_4_P_10_	SDX/**PYR**: 500/**25**	98.0 ± 4.6	C	110 ± 0	C
4_5_Z_1_	DHA/SDX/**PYR**: 60/500/**25**	103.5 ± 2.4	C	103 ± 0	C
4_6_Z_1_	DHA/SDX/**PYR**: 60/500/**25**	99.0 ± 5.1	C	99 ± 0	C
1_1_V_5_	**QUN: 50 mg/5 mL**	107.0 ± 3.7	C	109.7 ± 0.3	C
2_3_Z_1_	**DHA**/SDX/PYR: **60**/500/25	52.5 ± 9.5	NC	54.4 ± 0.2	NC
2_4_Z_1_	**DHA**/SDX/PYR: **60**/500/25	58.0 ± 7.8	NC	52.5 ± 0.1	NC
2_4_X_14_	ATM/**LUM**: 20/**120**	118.5 ± 3.8	NC	117.5 ± 0.8	NC
2X_15_	**ATM**/LUM: **20**/120	135.3 ± 3.7	NC	144.1 ± 0.7	NC
1_2_X_14_	**ATM**/LUM: **20**/120	35.0 ± 14.3	NC	35 ± 2	NC
4_3_Z_3_	**DHA**/PPQ: **40**/320	75.0 ± 10.0	NC	71 ± 3	NC
1_4_Z_3_	**DHA**/PPQ: **40**/320	75.0 ± 6.7	NC	70 ± 1	NC
4_1_R_8_	**QUN: 100 mg/5 mL**	137.5 ± 5.5	NC	291 ± 1	NC
1_2_P_2_	SDX/**PYR**: 500/**25**	74.0 ± 5.4	NC	76 ± 0	NC
4V_5_	**QUN: 50 mg/5 mL**	112.0 ± 4.5	BLC	112 ± 2	BLC

*Samples with different results from both assays*					

1_1_P_2_	**SDX**/PYR: **500**/25	86.0 ± 2.3	BLC	87 ± 2	NC
1_3_Z_1_	DHA/**SDX**/PYR: 60/**500**/25	88.0 ± 5.1	BLC	86 ± 2	NC
3_5_Z_1_	DHA/**SDX**/PYR: 60/**500**/25	88.5 ± 2.8	BLC	84.9 ± 0.1	NC
4_4_Y_12_	ATS/SM/**PYR**: 100/250/**12.5**	110.5 ± 4.5	BLC	119 ± 0	NC
2_5_X_11_	**ATM**/LUM: **80**/480	111.0 ± 6.8	BLC	113 ± 4	NC
4_3_Y_12_	ATS/SM/**PYR**: 100/250/**12.5**	115.0 ± 6.5	BLC	113 ± 0	NC
1_7_X_1_	**ATM**/LUM: **80**/480	89.0 ± 5.6	BLC	81.205 ± 0	NC
1_9_X_1_	ATM/**LUM**: 80/**480**	113.0 ± 4.0	BLC	113.5 ± 0.4	NC
3_3_P_10_	SDX/**PYR**: 500/**25**	88.0 ± 5.1	BLC	87 ± 0	NC
2_1_X_1_	ATM/**LUM**: 20/**120**	112.0 ± 4.0	BLC	113 ± 4	NC
1_2_Z_3_	**DHA**/PPQ: **40**/320	89.0 ± 5.6	BLC	86 ± 2	NC
1_2_V_5_	**QUN: 50 mg/5 mL**	111.0 ± 6.8	BLC	120 ± 1	NC
3_2_P_10_	SDX/**PYR**: 500/**25**	89.5 ± 5.0	BLC	94 ± 0	C
2_4_Y_13_	**ATS**/SDX/PYR: **100**/500/25	89.5 ± 5.0	BLC	97.57 ± 0.01	C
3_1_X_1_	**ATM**/LUM: **20**/120	89.5 ± 8.4	BLC	92.4 ± 0.6	C
1_3_V_5_	**QUN: 50 mg/5 mL**	109.0 ± 4.6	C	112 ± 1	BLC
4_2_Y_12_	ATS/SM/**PYR**: 200/500/**25**	106.0 ± 4.7	C	116 ± 0	NC
4_2_Z_3_	**DHA**/PPQ: **40**/320	97.5 ± 5.1	C	87 ± 1	NC
3_4_X_1_	ATM/**LUM**: 80/**480**	106.0 ± 6.6	C	111.6 ± 0.3	BLC
3_2_P_2_	SDX/**PYR**: 500/**25**	84.5 ± 5.3	NC	96 ± 0	C

**Table 4:** Comparison of quality of some antimalarial medicines by SQ-TLC and HPLC.

Code	Manufacturer's label claim (mg)	SQ-TLC results				HPLC results			
		Quality of individual API components in the order presented in label claim			Quality of the sample as a whole	Quality of individual API components in the order presented in label claim			Quality of the sample as a whole
2_4_Y_13_	ATS/SDX/PYR: 100/500/25	BLC	NC	C	**NC**	C	NC	C	**NC**
2X_15_	ATM/LUM: 20/120	NC	C		**NC**	NC	C		**NC**
2_4_X_14_	ATM/LUM: 20/120	NC	NC		**NC**	NC	NC		**NC**
1_2_X_14_	ATM/LUM: 20/120	NC	C		**NC**	NC	C		**NC**
2_3_Z_1_	DHA/SDX/PYR: 60/500/25	NC	C	C	**NC**	NC	C	C	**NC**
2_3_Z_1_	DHA/SDX/PYR: 60/500/25	NC	C	C	**NC**	NC	C	C	**NC**
1_4_P_10_	SDX/PYR: 500/25	NC	C		**NC**	NC	C		**NC**
1_3_Z_1_	DHA/SDX/PYR: 60/500/25	NC	BLC	C	**NC**	NC	NC	C	**NC**
3_5_Z_1_	DHA/SDX/PYR: 60/500/25	NC	BLC	C	**NC**	NC	NC	C	**NC**
1_6_Z_1_	DHA/PPQ: 40/320	C	—		**C**	C	—		**C**
1_1_V_5_	QUN: 50 mg/5 mL	C			**C**	C	—		**C**
4V_5_	QUN: 50 mg/5 mL	BLC			**BLC**	BLC			**BLC**
4_2_Y_12_	ATS/SM/PYR: 200/500/25	C	—	C	**C**	C	—	NC	**NC**
1_9_X_1_	ATM/LUM: 80/480	C	BLC		**BLC**	C	NC		**NC**
2_1_X_1_	ATM/LUM: 20/120	C	BLC		**BLC**	C	NC		**NC**
1_1_P_2_	SDX/PYR: 500/25	BLC	BLC		**BLC**	NC	NC		**NC**
1_2_Z_3_	DHA/PPQ: 40/320	BLC	—		**BLC**	NC	—		**NC**
1_2_V_5_	QUN: 50 mg/5 mL	BLC			**BLC**	NC			**NC**
1_3_V_5_	QUN: 50 mg/5 mL	C			**C**	BLC			**BLC**

**Table 5:** An estimated comparison of the cost drivers for HPLC and SQ-TLC analyses.

Cost factor	HPLC ($)	SQ-TLC ($)
Acquisition	≈100,000.00	Scanner/phone: 50.00
Stationery phase cost	≈490/100 injections = 4.9/sample. 4.9 × 6 = 29.4 per sample for 6 replicates	77/25 plates = 3.08/sample for 6 replicates
Solvent's cost	High (HPLC grade; highest purity)	Low (any grade)
Degassing	0.08/sample	Nil
Sample cleanup cost	0.78–15.51/sample	Nil
Annual maintenance	≈465–1485	Nil
Standby equipment and service technician	Yes	No
Precolumn	≈10–50	Nil
Validation	Needed frequently	Rarely needed
Wear and tear	High (due to high pressure)	Nil
Speed of analysis	Low	High
Cost per sample analysis	≈0.47–1.17	0.12–0.16
Parallel analysis	No, 1 at a time	Yes
Time per sample	2–60 minutes	5–10 minutes
Analyst skills needed	High to very high	Low to high
Analysis	Machine	Machine + eyes + software

## Data Availability

All data generated or analysed during this study are included in this published article and its supplementary information files.

## Supplementary Materials

Supplementary 1. HPLC and SQ-TLC results for active pharmaceutical ingredients.

Supplementary 2. HPLC and SQ-TLC results for antimalarial samples.
