# Supplementary material for: Further Validation of a Rapid Screening Semiquantitative Thin-Layer Chromatographic Method for Marketed Antimalarial Medicines for Adoption in Malawi
Source: J Anal Methods Chem. 2018 May 2;2018:2130390. doi: 10.1155/2018/2130390 (PMC5954873; doi:10.1155/2018/2130390)
Supplement: Supplementary 1. — HPLC and SQ-TLC results for active pharmaceutical ingredients. [file 2130390.f1.docx]

**SUPPLEMENTARY FILE 1**

**HPLC AND SQ-TLC RESULTS FOR ACTIVE PHARMACEUTICAL INGREDIENTS**

Table I: Percentage (%) and mass (mg) quantities of results of artesunate active pharmaceutical ingredient (API) by TLC and HPLC methods and their comparisons with the manufacturer’s claim and pharmacopoeial requirements. Artesunate tablets must contain at least 90.0% and at most 110.0% of the labelled amount of artesunate on the pack.

| **Code** | **Manufacturer’s**  **Label Claim (mg)** | **Semi-quantitative TLC estimation of composition in % and mg quantities of dosage forms of artesunate compared to the manufacturer’s label claim range of dosage forms (n = 6 for each solvent system, total n = 12)** | | | | | **Remarks based on TLC results** | **HPLC determination of composition of artesunate dosage forms in % and mg quantities (n = 6)** | | **Remarks**  **based on HPLC results** |
| --- | --- | --- | --- | --- | --- | --- | --- | --- | --- | --- |
|  |  | **Solvent system 1**  Ethanol : Ammonia  100 : 0.5 | | **Solvent system 2**  Ethanol: Toluene: Ammonia  70 : 30 : 1.5 | | **%Average ± rsd** |  |  |  |  |
|  |  | % range ± sd | Quantity (mg) | % range ± sd | Quantity (mg) |  |  | % ± rsd | Quantity(mg) |  |
| *2_4_Y_13_ | **ATS**/SDX/PYR:**100**/500/25 | 87-97±5 | 87-97 | 82-92 ± 4 | 82-92 | 89.5 ± 5.0 | **BLC** | 97.57 ± 0.01 | 98 | **C** |
| *4Y_13_ | **ATS**/SDX/PYR:**100**/500/25 | 90-100± 5 | 90-100 | 92-102 ± 4 | 92-102 | 96.0 ± 4.7 | **C** | 94.8 ± 0.1 | 95 | **C** |
| *3_4_Y_13_ | **ATS**/SDX/PYR:**100**/500/25 | 100-110 ± 5 | 100-110 | 89-99 ± 5 | 89-99 | 99.5 ± 5.0 | **C** | 97.65 ± 0.08 | 98 | **C** |
| *3_2_Y_13_ | **ATS**/SDX/PYR:**100**/500/25 | 90-100 ± 5 | 90-100 | 87-97 ± 5 | 87-97 | 93.5 ± 5.3 | **C** | 98.11 ± 0.02 | 98 | **C** |
| 4_4_Y_12_ | **ATS**/SM/PYR:**100**/250/12 | 90-100 ± 10 | 90-100 | 95-105 ± 10 | 95-105 | 97.5 ± 10.3 | **C** | 90.4 ± 0.2 | 90 | C |
| 4_2_Y_12_ | **ATS**/SM/PYR:**200**/500/25 | 87-97 ± 5 | 174-194 | 85-95 ± 5 | 170-190 | 91.0 ± 5.5 | **C** | 92.77 ± 0.02 | 186 | C |
| 4_3_Y_12_ | **ATS**/SM/PYR:**100**/250/12.5 | 65-75 ± 10 | 65-75 | 70-80 ± 10 | 70-80 | 72.5 ± 13.8 | **NC** | 78.22 ± 0.04 | 78 | **NC** |
| 4_1_Y_12_ | **ATS**/SM/PYR:**200**/500/25 | 90-100 ± 5 | 180-200 | 89-99 ± 5 | 178-198 | 94.5 ± 5.3 | **C** | 86.9 ± 0.2 | 174 | **NC** |
| 3_1_Y_12_ | **ATS**/SM/PYR:**200**/500/25 | 65-75 ± 10 | 130-150 | 77-87 ± 5 | 154-174 | 76.0 ± 9.9 | **NC** | 88.96 ± 0.05 | 178 | **BLC** |

Summary - 66.67% (6/9) samples gave the same conclusion for both assays

Table II: Percentage (%) and mass (mg) quantities of results of artemether active pharmaceutical ingredient (API) by TLC and HPLC methods and their comparisons with the manufacturer’s claim and pharmacopoeial requirements. Artemether tablets must contain at least 90.0% and at most 110.0% of the labelled amount of artemether on the pack

| **Code** | **Manufacturer’s**  **Label Claim (mg)** | **Semi-quantitative TLC estimation of composition in % and mg quantities of dosage forms of artemether compared to the manufacturer’s label claim ( n = 6 for each solvent system, total n = 12)** | | | | | **Remarks based on TLC results** | **HPLC determination of composition of artemether dosage forms in % and mg quantities (n = 6)** | | **Remarks based on HPLC results** |
| --- | --- | --- | --- | --- | --- | --- | --- | --- | --- | --- |
|  |  | **Solvent system 1**  Petrol: Ethyl acetate  70 : 30 | | **Solvent system 2**  Petrol: Ethyl acetate  60 : 40 | | **% Average ± rsd** |  |  |  |  |
|  |  | % range ± sd | Quantity (mg) | % range ± sd | Quantity (mg) |  |  | % ± rsd | Quantity (mg) |  |
| 2X_15_ | **ATM**/LUM:**20**/120 | 127-137 ± 5 | 25-27 | 134-144 ± 5 | 27-29 | 135.3 ± 3.7 | **NC** | 144.1 ± 0.7 | 29 | **NC** |
| 4X_20_ | **ATM**/LUM:**20**/120 | 95-105 ± 5 | 19-21 | 92-102 ± 4 | 18-20 | 98.5 ± 4.6 | **C** | 100 ± 2 | 20 | **C** |
| 1_1_X_1_ | **ATM**/LUM:**20**/120 | 59-69 ± 4 | 12-14 | 55-65 ± 5 | 11-13 | 62.0 ± 7.3 | **NC** | 58.9 ± 0.6 | 12 | **NC** |
| 1_1_X_11_ | **ATM**/LUM:**180**/1080 | 97-107 ± 5 | 175-193 | 99-109 ± 4 | 178-196 | 103.0 ± 4.4 | **C** | 93 ± 2 | 167 | **C** |
| 1_1_X_14_ | **ATM**/LUM:**20**/120 | 79-89 ± 4 | 16-18 | 78-88 ± 4 | 16-18 | 83.5 ± 4.8 | **NC** | 75.3 ± 0.9 | 15 | **NC** |
| 1_1_X_17_ | **ATM**/LUM:**80**/480 | 115-125 ± 15 | 92-100 | 125-135± 15 | 100-108 | 125.0 ± 12.0 | **NC** | 177.8 ± 0.3 | 142 | **NC** |
| 1_1_X_18_ | **ATM**/LUM:**40**/240 | 30-40 ± 5 | 12-16 | 29-39 ± 4 | 12-16 | 34.5 ± 13.0 | **NC** | 26.5 ± 0.8 | 11 | **NC** |
| 1_1_X_20_ | **ATM**/LUM:**20**/120 | 132-142 ± 4 | 26-28 | 134-144 ± 5 | 27-29 | 138.0 ± 3.3 | **NC** | 135 ± 4 | 27 | **NC** |
| 1_2_X_1_ | **ATM**/LUM:**20**/120 | 119-129 ± 4 | 24-26 | 115-125 ± 5 | 23-25 | 122.0 ± 3.7 | **NC** | 119.7 ± 0.8 | 24 | **NC** |
| 1_2_X_11_ | **ATM**/LUM:**180**/1080 | 105-115 ± 5 | 189-207 | 100-110 ± 5 | 180-198 | 107.5 ± 4.7 | **C** | 103.3 ± 0.8 | 186 | **C** |
| 1_2_X_14_ | **ATM**/LUM:**20**/120 | 30-40 ± 5 | 6-8 | 30-40 ± 5 | 6-8 | 35.0 ± 14.3 | **NC** | 35 ± 2 | 7 | **NC** |
| 1_3_X_1_ | **ATM**/LUM:**40**/240 | 92-102 ± 4 | 37-41 | 94-104 ± 5 | 38-42 | 98.0 ± 4.6 | **C** | 96.8 ± 0.2 | 39 | **C** |
| 1_3_X_11_ | **ATM**/LUM:**80**/480 | 35-45 ± 5 | 28-36 | 35-45 ± 5 | 28-36 | 40.0 ± 12.5 | **NC** | 39.6 ± 0.5 | 32 | **NC** |
| 1_4_X_1_ | **ATM**/LUM:**40**/240 | 64-74 ± 5 | 26-30 | 67-77 ± 5 | 27-31 | 70.5 ± 7.1 | **NC** | 69 ± 2 | 28 | **NC** |
| 1_4_X_11_ | **ATM**/LUM:**40**/240 | 75-85 ± 5 | 30-34 | 72-82 ± 4 | 29-33 | 78.5 ± 5.7 | **NC** | 82.7 ± 0.3 | 33 | **NC** |
| 1_5_X_1_ | **ATM**/LUM:**80**/480 | 65-75 ± 5 | 52-60 | 62-72 ± 4 | 50-58 | 68.5 ± 6.6 | **NC** | 67.3 ± 0.3 | 54 | **NC** |
| 1_5_X_11_ | **ATM**/LUM:**20**/120 | 40-50 ± 0 | 8-10 | 40-50 ± 0 | 8-10 | 45.0 ± 0.0 | **NC** | 45 ± 1 | 9 | **NC** |
| 1_6_X_1_ | **ATM**/LUM:**40**/240 | 125-135 ± 10 | 50-54 | 120-130 ± 5 | 48-52 | 127.5 ± 5.9 | **NC** | 163.2 ± 0.4 | 65 | **NC** |
| 1_7_X_1_ | **ATM**/LUM:**80**/480 | 84-94 ± 5 | 67-75 | 84-94 ± 5 | 67-75 | 89.0 ± 5.6 | **BLC** | 81.205±0 | 65 | **NC** |
| 1_8_X_1_ | **ATM**/LUM:**20**/120 | 120-130 ± 5 | 24-26 | 120-130 ± 5 | 24-26 | 125.0 ± 4.0 | **NC** | 162.7 ± 0.3 | 33 | **NC** |
| 1_9_X_1_ | **ATM**/LUM:**80**/480 | 105-115 ± 5 | 84-92 | 105-115 ± 5 | 84-92 | 110.0 ± 4.5 | **C** | 103.4 ± 0.4 | 83 | **C** |
| 2_1_X_1_ | **ATM**/LUM:**20**/120 | 97-107± 5 | 19-21 | 97-107 ± 5 | 19-21 | 102.0 ± 4.9 | **C** | 97 ± 1 | 19 | **C** |
| 2_2_X_1_ | **ATM**/LUM:**80**/480 | 120-130 ± 5 | 96-104 | 115-125± 15 | 92-100 | 122.5 ± 8.2 | **NC** | 131.5 ± 0.2 | 105 | **NC** |
| 2_3_X_1_ | **ATM**/LUM:**20**/120 | 135-145 ± 5 | 27-29 | 129-139 ± 4 | 26-28 | 137.0 ± 3.3 | **NC** | 149 ± 1 | 30 | **NC** |
| 2_4_X_14_ | **ATM**/LUM:**20**/120 | 52-62 ± 4 | 10-12 | 50-60 ± 0 | 10-12 | 56.0 ± 3.6 | **NC** | 53.2 ± 0.3 | 11 | **NC** |
| 2_5_X_11_ | **ATM**/LUM:**80**/480 | 105-115 ± 10 | 84-92 | 107-117 ± 5 | 86-94 | 111.0 ± 6.8 | **BLC** | 113 ± 4 | 90 | **NC** |
| 3_1_X_1_ | **ATM**/LUM:**20**/120 | 85-95 ± 10 | 17-19 | 84-94 ± 5 | 17-19 | 89.5 ± 8.4 | **BLC** | 92.4 ± 0.6 | 18 | **C** |
| 3_2_X_1_ | **ATM**/LUM:**20**/120 | 92-102 ± 4 | 18-20 | 95-105 ± 5 | 19-21 | 98.5 ± 4.6 | **C** | 97 ± 1 | 19 | **C** |
| 3_1_X_11_ | **ATM**/LUM:**80**/480 | 32-42 ± 4 | 26-34 | 34-44 ± 5 | 27-35 | 38.0 ± 11.8 | **NC** | 39 ± 1 | 31 | **NC** |
| 3_3_X_1_ | **ATM**/LUM:**40**/240 | 74-84 ± 5 | 30-34 | 77-87 ± 5 | 31-35 | 80.5 ± 6.2 | **NC** | 81.0 ± 0.3 | 32 | **NC** |
| 3_4_X_1_ | **ATM**/LUM:**80**/480 | 95-105 ± 5 | 76-84 | 95-105 ± 5 | 76-84 | 100.0 ± 5.0 | **C** | 102.1 ± 0.7 | 82 | **C** |
| 3_6_X_1_ | **ATM**/LUM:**80**/480 | 99-109 ± 4 | 79-87 | 95-105 ± 5 | 76-84 | 102.0 ± 4.4 | **C** | 104.8 ± 0.3 | 84 | **C** |
| 4_1_X_11_ | **ATM**/LUM:**20**/120 | 105-115 ± 5 | 21-23 | 100-110± 10 | 20-22 | 107.5 ± 6.9 | **C** | 106.2 ± 0.4 | 21 | **C** |
| 4_2_X_11_ | **ATM**/LUM:**20**/120 | 24-34 ± 5 | 5-7 | 25-35 ± 5 | 5-7 | 29.5 ± 16.9 | **NC** | 28 ± 1 | 6 | **NC** |
| 4_3_X_1_ | **ATM**/LUM:**40**/240 | 132-142 ± 4 | 53-57 | 130-140 ± 0 | 52-56 | 136.0 ± 1.5 | **NC** | 173.8 ± 0.3 | 70 | **NC** |
| 4_4_X_1_ | **ATM**/LUM:**80**/480 | 135-145 ± 5 | 108-116 | 135-145± 10 | 108-116 | 140.0 ± 5.4 | **NC** | 142.6 ± 0.7 | 114 | **NC** |
| 4_5_X_12_ | **ATM**/LUM:**180**/1080 | 90-100 ± 5 | 162-180 | 90-100 ± 5 | 162-180 | 95.0 ± 5.3 | **C** | 96.0 ± 0.9 | 173 | **C** |
| 1_10_X_1_ | **ATM**/LUM:**80**/480 | 120-130 ± 5 | 96-104 | 124-134 ± 5 | 99-107 | 127.0 ± 3.9 | **NC** | 124.2 ± 0.5 | 99 | **NC** |
| 1_6_X_11_ | **ATM**/LUM:**180**/1080 | 100-110 ± 5 | 180-198 | 100-110 ± 5 | 180-198 | 105.0 ± 4.8 | **C** | 99.3 ± 0.9 | 179 | **C** |
| 1_12_X_1_ | **ATM**/LUM:**80**/480 | 92-102 | 74-82 | 90-100 ± 5 | 72-80 | 96.0 ± 2.6 | **C** | 100.9 ± 0 | 81 | **C** |
| 1_13_X_1_ | **ATM**/LUM:**80**/480 | 112-122 ± 4 | 90-98 | 115-125 ± 5 | 92-100 | 118.0 ± 3.8 | **NC** | 140.0 ± 0.5 | 112 | **NC** |

Summary – 92.68% (38/41) samples gave the same conclusion for both assays

Table III: Percentage (%) and mass (mg) quantities of results of lumefantrine active pharmaceutical ingredient (API) by TLC and HPLC methods and their comparisons with the manufacturer’s claim and pharmacopoeial requirements. Lumefantrine tablets must contain at least 90.0% and at most 110.0% of the labelled amount of Lumefantrine on the pack

| **Code** | **Manufacturer’s**  **Label Claim (mg)** | **Semi-quantitative TLC estimation of composition in % and mg quantities of dosage forms of lumefantrine compared to the manufacturer’s label claim ( n = 6 for each solvent system, total n = 12)** | | | | | **Remarks based on TLC results** | **HPLC determination of composition of lumefantrine dosage forms in % and mg quantities**  **(n = 6)** | | **Remarks based on HPLC results** |
| --- | --- | --- | --- | --- | --- | --- | --- | --- | --- | --- |
|  |  | **Solvent system 1**  Ethyl acetate: acetic acid: toluene  4:2:18 | | **Solvent system 2**  Ethyl acetate: acetic acid  10:5 | | **% Average ± rsd** |  |  |  |  |
|  |  | % range ± sd | Quantity (mg) | % range ± sd | Quantity (mg) |  |  | % ± rsd | Quantity (mg) |  |
| 2X_15_ | ATM/**LUM**:20/**120** | 95-105 ± 5 | 114-126 | 95-105 ± 5 | 114-126 | 100.0 ± 5.0 | **C** | 97 ± 2 | 116 | **C** |
| 4X_20_ | ATM/**LUM**:20/**120** | 112-122± 4 | 134-146 | 113-123±5 | 136-148 | 117.5 ± 3.8 | **NC** | 119.5 ± 0.4 | 143 | **NC** |
| 1_1_X_1_ | ATM/**LUM**:20/**120** | 104-114± 5 | 125-137 | 95-105 ± 5 | 114-126 | 104.5 ± 4.8 | **C** | 112.5 ± 0.5 | 135 | **NC** |
| 1_1_X_11_ | ATM/**LUM**:180/**1080** | 117-127± 5 | 1264-1372 | 120-130 ± 0 | 1296-1404 | 123.5 ± 2.0 | **NC** | 127 ± 1 | 1372 | **NC** |
| 1_1_X_14_ | ATM/**LUM**:20/**120** | 60-70 ± 0 | 72-84 | 60-70 ± 0 | 72-84 | 65.5 ± 0 | **NC** | 69.3 ± 0.3 | 83 | **NC** |
| 1_1_X_17_ | ATM/**LUM**:80/**480** | 94-104 ± 5 | 451-499 | 98-108 ± 5 | 470-518 | 101.0 ± 5.0 | **C** | 101.0 ± 0.4 | 485 | **C** |
| 1_1_X_18_ | ATM/**LUM**:40/**240** | 95-105 ± 5 | 228-252 | 90-100 ± 0 | 216-240 | 97.5 ± 2.6 | **C** | 92.9 ± 0.3 | 223 | **C** |
| 1_1_X_20_ | ATM/**LUM**:20/**120** | 109-119± 4 | 131-143 | 110-120 ± 0 | 132-144 | 114.5 ± 1.7 | **BLC** | 114.5 ± 0.5 | 137 | **NC** |
| 1_2_X_1_ | ATM/**LUM**:20/**120** | 100-110 ± 5 | 120-132 | 94-104 ± 5 | 113-125 | 102.0 ± 5 | **C** | 113.0 ± 0.4 | 136 | **NC** |
| 1_2_X_11_ | ATM/**LUM**:180/**1080** | 122-132± 4 | 1318-1426 | 119-129 ± 4 | 1285-1393 | 125.5 ± 3.2 | **NC** | 123 ± 2 | 1328 | **NC** |
| 1_2_X_14_ | ATM/**LUM**:20/**120** | 92-102 ± 4 | 110-122 | 90-100 ± 5 | 108-120 | 96.0 ± 4.7 | **C** | 95.3 ± 0.2 | 114 | **C** |
| 1_3_X_1_ | ATM/**LUM**:40/**240** | 77-87 ± 5 | 185-209 | 74-84 ± 5 | 178-202 | 80.5 ± 6.2 | **NC** | 81.1 ± 0.3 | 195 | **NC** |
| 1_3_X_11_ | ATM/**LUM**:80/**480** | 74-84 ± 5 | 355-403 | 75-85 ± 5 | 360-408 | 79.5 ± 6.3 | **NC** | 85 ± 1 | 408 | **NC** |
| 1_4_X_1_ | ATM/**LUM**:40/**240** | 100-110 ± 5 | 240-264 | 109-119 ± 4 | 262-286 | 109.5 ± 4.1 | **C** | 115.0 ± 0.9 | 276 | **NC** |
| 1_4_X_11_ | ATM/**LUM**:40/**240** | 100-110 ± 5 | 240-264 | 105-115 ± 5 | 252-276 | 107.5 ± 4.7 | **C** | 114.0 ± 0.4 | 274 | **NC** |
| 1_5_X_1_ | ATM/**LUM**:80/**480** | 99-109 ± 4 | 475-523 | 103-113 ± 5 | 494-542 | 106.0 ± 4.2 | **C** | 110.9 ± 0.2 | 532 | **BLC** |
| 1_5_X_11_ | ATM/**LUM**:20/**120** | 80-90 ± 10 | 96-108 | 90-100 ± 5 | 108-120 | 90.0 ± 8.3 | **C** | 90.5 ± 0.3 | 109 | **C** |
| 1_6_X_1_ | ATM/**LUM**:40/**240** | 122-132± 4 | 293-317 | 120-130 ± 10 | 288-312 | 126.0 ± 5.6 | **NC** | 127 ± 1 | 305 | **NC** |
| 1_7_X_1_ | ATM/**LUM**:80/**480** | 57-67 ± 5 | 274-322 | 59-69 ± 4 | 283-331 | 63.0 ± 7.1 | **NC** | 59 ± 1 | 283 | **NC** |
| 1_8_X_1_ | ATM/**LUM**:20/**120** | 70-80 ± 0 | 84-96 | 72-82 ± 4 | 86-98 | 76.0 ± 2.6 | **NC** | 79.19 ± 0.0 | 95 | **NC** |
| 1_9_X_1_ | ATM/**LUM**:80/**480** | 107-117± 5 | 514-562 | 109-119 ± 4 | 523-571 | 113.0 ± 4.0 | **BLC** | 113.5 ± 0.4 | 545 | **NC** |
| 2_1_X_1_ | ATM/**LUM**:20/**120** | 105-115 ± 5 | 126-138 | 109-119 ± 4 | 131-143 | 112.0 ± 4.0 | **BLC** | 113 ± 4 | 136 | **NC** |
| 2_2_X_1_ | ATM/**LUM**:80/**480** | 57-67 ± 5 | 274-322 | 60-70 ± 0 | 288-336 | 63.5 ± 3.9 | **NC** | 63 ± 2 | 302 | **NC** |
| 2_3_X_1_ | ATM/**LUM**:20/**120** | 97-107 ± 5 | 116-128 | 99-109 ± 4 | 119-131 | 103.0 ± 4.4 | **C** | 103.3 ± 0.3 | 124 | **C** |
| 2_4_X_14_ | ATM/**LUM**:20/**120** | 112-122± 4 | 134-146 | 115-125 ± 5 | 138-150 | 118.5 ± 3.8 | **NC** | 117.5 ± 0.8 | 141 | **NC** |
| 2_5_X_11_ | ATM/**LUM**:80/**480** | 90-100 ± 10 | 432-480 | 100-110 ± 5 | 480-528 | 100.0 ± 7.5 | **C** | 112.2 ± 0.6 | 539 | **NC** |
| 3_1_X_1_ | ATM/**LUM**:20/**120** | 115-125 ± 5 | 138-150 | 119-129 ± 4 | 143-155 | 122.0 ± 3.7 | **NC** | 116.9 ± 0.5 | 140 | **NC** |
| 3_2_X_1_ | ATM/**LUM**:20/**120** | 112-122± 4 | 134-146 | 112-122 ± 4 | 134-146 | 117.0 ± 3.4 | **NC** | 117 ± 2 | 140 | **NC** |
| 3_1_X_11_ | ATM/**LUM**:80/**480** | 100-110 ± 10 | 480-528 | 105-115 ± 10 | 504-552 | 107.5 ± 9.3 | **C** | 118.6 ± 0.6 | 569 | **NC** |
| 3_3_X_1_ | ATM/**LUM**:40/**240** | 95-105 ± 5 | 228-252 | 95-105 ± 5 | 228-252 | 100.0 ± 5.0 | **C** | 108.0 ± 0.2 | 259 | **C** |
| 3_4_X_1_ | ATM/**LUM**:80/**480** | 100-110 ± 10 | 480-528 | 102-112 ± 4 | 490-538 | 106.0 ± 6.6 | **C** | 111.6 ± 0.3 | 536 | **BLC** |
| 3_6_X_1_ | ATM/**LUM**:80/**480** | 90-100 ± 5 | 432-480 | 95-105 ± 5 | 456-504 | 97.5 ± 5.1 | **C** | 103.3 ± 0.1 | 496 | **C** |
| 4_1_X_11_ | ATM/**LUM**:20/**120** | 45-55 ± 5 | 54-66 | 40-50 ± 0 | 48-60 | 47.5 ± 5.3 | **NC** | 50 ± 6 | 60 | **NC** |
| 4_2_X_11_ | ATM/**LUM**:20/**120** | 87-97 ± 5 | 104-116 | 90-100 ± 5 | 108-120 | 93.5 ± 5.3 | **C** | 88 ± 2 | 106 | **BLC** |
| 4_3_X_1_ | ATM/**LUM**:40/**240** | 72-82 ± 4 | 173-197 | 75-85 ± 5 | 180-204 | 78.5 ± 5.7 | **NC** | 81.8 ± 0.3 | 196 | **NC** |
| 4_4_X_1_ | ATM/**LUM**:80/**480** | 100-110 ± 5 | 480-528 | 95-105 ± 5 | 456-504 | 102.5 ± 4.9 | **C** | 104.0 ± 0.2 | 499 | **C** |
| 4_5_X_12_ | ATM/**LUM**:180/**1080** | 125-135 ± 5 | 1350-1458 | 125-135 ± 5 | 1350-1458 | 130.0 ± 3.8 | **NC** | 129 ± 2 | 1393 | **NC** |
| 1_10_X_1_ | ATM/**LUM**:80/**480** | 62-72 ± 4 | 298-346 | 60-70 ± 0 | 288-336 | 66.0 ± 3.0 | **NC** | 63 ± 1 | 302 | **NC** |
| 1_6_X_11_ | ATM/**LUM**:180/**1080** | 107-117± 5 | 1156-1264 | 108-118 ± 5 | 1166-1274 | 112.5 ± 4.4 | **BLC** | 119.3 ± 0.1 | 1288 | **NC** |
| 1_12_X_1_ | ATM/**LUM**:80/**480** | 100-110 ± 5 | 480-528 | 94-104 ± 5 | 451-499 | 102.0 ± 4.9 | **C** | 102.5 ± 0 | 492 | **C** |
| 1_13_X_1_ | ATM/**LUM**:80/**480** | 90-100 ± 5 | 432-480 | 95-105 ± 5 | 456-504 | 97.5 ± 5.1 | **C** | 109.96±0.0 | 528 | **C** |

Summary - 68.29% (28/41) samples gave the same conclusion for both assays

Table IV: Percentage (%) and mass (mg) quantities of results of dihydroartemisinin (Artenimol) active pharmaceutical ingredient (API) by TLC and HPLC methods and their comparisons with the manufacturer’s claim and pharmacopoeial requirements. Dihydroartemisinin tablets must contain at least 90.0% and at most 110.0% of the labelled amount of Dihydroartemisinin on the pack

| **Code** | **Manufacturer’s**  **Label Claim (mg)** | **Semi-quantitative TLC estimation of composition in % and mg quantities of dosage forms of dihydroartemisinin compared to the manufacturer’s label claim (n = 6 for each solvent system, total n = 12)** | | | | | **Remarks based on TLC results** | **HPLC determination of composition of dihydroartemisinin dosage forms in % and mg quantities**  **(n = 6)** | | **Remarks based on HPLC results** |
| --- | --- | --- | --- | --- | --- | --- | --- | --- | --- | --- |
|  |  | **Solvent system 1**  Toluene: Ethyl acetate  60 : 40 | | **Solvent system 2**  Toluene: Ethyl acetate  70 : 30 | | **% Average ± rsd** |  |  |  |  |
|  |  | % range ± sd | Quantity  (mg) | % range ± sd | Quantity (mg) |  |  | % ± rsd | Quantity (mg) |  |
| 1_1_Z_3_ | **DHA**/PPQ:**40**/320 | 100-110 ± 5 | 40-44 | 95-105 ± 5 | 38-42 | 102.5 ± 4.9 | **C** | 98.7 ± 0.2 | 39 | **C** |
| 1_2_Z_3_ | **DHA**/PPQ:**40**/320 | 84-94± 5 | 34-38 | 84-94± 5 | 34-38 | 89.0 ± 5.6 | **BLC** | 86 ± 2 | 34 | **NC** |
| 1_4_Z_3_ | **DHA**/PPQ:**40**/320 | 70-80 ± 5 | 28-32 | 70-80 ± 5 | 28-32 | 75.0 ± 6.7 | **NC** | 70 ± 1 | 28 | **NC** |
| 1_6_Z_1_ | **DHA**/PPQ:**40**/320 | 90-100 ± 10 | 36-40 | 94-104± 5 | 38-42 | 97.0 ± 7.7 | **C** | 97.5 ± 0.9 | 39 | **C** |
| 1_7_Z_1_ | **DHA**/PPQ:**40**/320 | 85-95 ± 10 | 34-38 | 85-95 ± 10 | 34-38 | 90.0 ± 11.0 | **C** | 85 ± 1 | 34 | **NC** |
| 2_6_Z_1_ | **DHA**/PPQ:**40**/320 | 95-105 ± 10 | 38-42 | 95-105 ± 10 | 38-42 | 100.0 ± 10.0 | **C** | 103 ± 1 | 41 | **C** |
| 2_7_Z_1_ | **DHA**/PPQ:**40**/320 | 102-112± 4 | 41-45 | 100-110 ± 5 | 40-44 | 106.0 ± 4.2 | **C** | 88 ± 5 | 35 | **BLC** |
| 3_2_Z_3_ | **DHA**/PPQ:**40**/320 | 67-77 ± 5 | 27-31 | 66-76 ± 5 | 26-30 | 71.5 ± 7.0 | **NC** | 73 ± 2 | 29 | **NC** |
| 3_3_Z_3_ | **DHA**/PPQ:**40**/320 | 62-72 ± 4 | 25-29 | 64-74 ± 5 | 26-30 | 68.0 ± 6.6 | **NC** | 71 ± 1 | 28 | **NC** |
| 3_7_Z_1_ | **DHA**/PPQ:**40**/320 | 90-100 ± 5 | 36-40 | 95-105 ± 5 | 38-42 | 97.5 ± 5.1 | **C** | 101 ± 6 | 40 | **C** |
| 4_1_Z_3_ | **DHA**/PPQ:**40**/320 | 69-79 ± 5 | 28-32 | 67-77 ± 5 | 27-31 | 73.0 ± 6.8 | **NC** | 74 ± 3 | 30 | **NC** |
| 4_2_Z_3_ | **DHA**/PPQ:**40**/320 | 90-100 ± 10 | 36-40 | 95-105 ± 5 | 38-42 | 97.5 ± 7.7 | **C** | 87 ± 1 | 35 | **NC** |
| 4_3_Z_3_ | **DHA**/PPQ:**40**/320 | 70-80 ± 5 | 28-32 | 70-80 ± 10 | 28-32 | 75.0 ± 10.0 | **NC** | 71 ± 3 | 28 | **NC** |
| 4_4_Z_3_ | **DHA**/PPQ:**40**/320 | 90-100 ± 5 | 36-40 | 90-100 ± 5 | 36-40 | 95.0 ± 5.3 | **C** | 88 ± 1 | 35 | **BLC** |
| 1_5_Z_1_ | **DHA**/SDX/PYR:**60**/500/25 | 47-57 ± 5 | 28-34 | 45-55 ± 5 | 27-33 | 51.0 ± 9.8 | **NC** | 51.4 ± 0.2 | 31 | **NC** |
| 1_3_Z_1_ | **DHA**/SDX/PYR:**60**/500/25 | 56-66 ± 4 | 34-40 | 60-70 ± 5 | 36-42 | 63.0 ± 7.1 | **NC** | 56.8 ± 0.2 | 34 | **NC** |
| 1_2_Z_1_ | **DHA**/SDX/PYR:**60**/500/25 | 57-67 ± 5 | 34-40 | 57-67 ± 5 | 34-40 | 62.0 ± 8.1 | **NC** | 52.5 ± 0.1 | 32 | **NC** |
| 1_4_Z_1_ | **DHA**/SDX/PYR:**60**/500/25 | 60-70 ± 5 | 36-42 | 55-65 ± 5 | 33-39 | 62.5 ± 8.0 | **NC** | 61.6 ± 0.1 | 37 | **NC** |
| 2_1_Z_1_ | **DHA**/SDX/PYR:**60**/500/25 | 60-70 ± 5 | 36-42 | 60-70 ± 5 | 36-42 | 65.0 ± 7.7 | **NC** | 56.48±0.09 | 34 | **NC** |
| 2_3_Z_1_ | **DHA**/SDX/PYR:**60**/500/25 | 45-55 ± 5 | 27-33 | 50-60 ± 5 | 30-36 | 52.5 ± 9.5 | **NC** | 54.4 ± 0.2 | 33 | **NC** |
| 2_4_Z_1_ | **DHA**/SDX/PYR:**60**/500/25 | 54-64 ± 5 | 32-38 | 52-62 ± 4 | 31-37 | 58.0 ± 7.8 | **NC** | 52.5 ± 0.1 | 32 | **NC** |
| 2_5_Z_1_ | **DHA**/SDX/PYR:**60**/500/25 | 55-65 ± 5 | 33-39 | 57-67 ± 5 | 34-40 | 61.0 ± 8.2 | **NC** | 51.25±0.06 | 31 | **NC** |
| 3_4_Z_1_ | **DHA**/SDX/PYR:**60**/500/25 | 49-59 ± 4 | 29-35 | 49-59 ± 4 | 29-35 | 54.0 ± 7.4 | **NC** | 51.4 ± 0.1 | 31 | **NC** |
| 3_5_Z_1_ | **DHA**/SDX/PYR:**60**/500/25 | 56-66 ± 5 | 34-40 | 52-62 ± 4 | 31-37 | 59.0 ± 7.6 | **NC** | 51.8 ± 0.1 | 31 | **NC** |
| 4_5_Z_1_ | **DHA**/SDX/PYR:**60**/500/25 | 57-67 ±5 | 34-40 | 57-67±5 | 34-40 | 62.0 ± 8.1 | **NC** | 51.67±0.05 | 31 | **NC** |
| 4_6_Z_1_ | **DHA**/SDX/PYR:**60**/500/25 | 57-67± 5 | 34-40 | 55-65 ± 10 | 33-39 | 61.0 ± 12.3 | **NC** | 51.92±0.09 | 31 | **NC** |

Summary - 80.77% (21/26) samples gave the same conclusion for both assays

Table V: Percentage (%) and Mass (mg) quantities of results of sulphadoxine/sulphamethoxypyridazine active pharmaceutical ingredient (API) by TLC and HPLC methods and their comparisons with the manufacturer’s claim and pharmacopoeial requirements. Sulphadoxine tablets must contain at least 90.0% and at most 110.0% of the labelled amount of Sulphadoxine on the pack

| **Code** | **Manufacturer’s**  **Label Claim (mg)** | **Semi-quantitative TLC estimation of composition in % and mg quantities of dosage forms of sulphadoxine compared to the manufacturer’s label claim n = 6 for each solvent system, total n=12)** | | | | | **Remarks based on TLC results** | **HPLC determination of composition of sulphadoxine dosage forms in % and mg quantities (n = 6)** | | **Remarks based on HPLC results** |
| --- | --- | --- | --- | --- | --- | --- | --- | --- | --- | --- |
|  |  | **Solvent system 1**  Ethyl acetate/methanol/  Ammonia 80:15:5 | | **Solvent system 2**  Ethyl acetate/acetic acid/  water 60:20:20 | | **% Average ± rsd** |  |  |  |  |
|  |  | % range ± sd | Quantity (mg) | % range ± sd | Quantity (mg) |  |  | % ± rsd | Quantity (mg) |  |
| 1_1_P_10_ | **SDX**/PYR:**500**/25 | 80-90 ± 5 | 400-450 | 77-87±5 | 385-435 | 83.5 ± 6.0 | **NC** | 78 ± 2 | 390 | **NC** |
| 1_1_P_2_ | **SDX**/PYR:**500**/25 | 82-92±4 | 410-460 | 80-90 ± 0 | 400-450 | 86.0 ± 2.3 | **BLC** | 87 ± 2 | 435 | **NC** |
| 1_2_P_2_ | **SDX**/PYR:**500**/25 | 70-80 ± 5 | 350-400 | 70-80 ± 5 | 350-400 | 75.0 ± 6.7 | **NC** | 67 ± 4 | 335 | **NC** |
| 1_4_P_10_ | **SDX**/PYR:**500**/25 | 74-84± 5 | 370-420 | 77-87 ± 5 | 385-435 | 80.5 ± 6.2 | **NC** | 85 ± 2 | 425 | **NC** |
| 1_4_P_2_ | **SDX**/PYR:**500**/25 | 79-89± 4 | 395-445 | 78-88 ± 5 | 390-440 | 83.5 ± 5.4 | **NC** | 82 ± 2 | 410 | **NC** |
| 1_6_P_10_ | **SDX**/PYR:**500**/25 | 82-92± 4 | 410-460 | 80-90 ± 5 | 400-450 | 86.0 ± 5.2 | **BLC** | 83 ± 3 | 415 | **NC** |
| 2_1_P_15_ | **SDX**/PYR:**500**/25 | 77-87± 5 | 385-435 | 78-88 ± 4 | 390-440 | 82.8 ± 5.4 | **NC** | 82 ± 3 | 410 | **NC** |
| 2_1_P_2_ | **SDX**/PYR:**500**/25 | 87-97± 5 | 435-485 | 80-90 ± 0 | 400-450 | 88.5 ± 2.8 | **BLC** | 77 ± 1 | 385 | **NC** |
| 2_2_P_2_ | **SDX**/PYR:**500**/25 | 85-95 ± 5 | 425-475 | 83-93 ± 5 | 415-465 | 89.0 ± 5.6 | **BLC** | 83 ± 2 | 415 | **NC** |
| 2_3_P_2_ | **SDX**/PYR:**500**/25 | 90-100± 5 | 450-500 | 90-100 ± 0 | 450-500 | 95.0 ± 2.6 | **C** | 96 ± 2 | 480 | **C** |
| 3_1_P_10_ | **SDX**/PYR:**500**/25 | 94-104±5 | 470-520 | 85-95 ± 5 | 425-475 | 94.5 ± 5.3 | **C** | 83 ± 2 | 415 | **NC** |
| 3_1_P_2_ | **SDX**/PYR:**500**/25 | 94-104± 5 | 470-520 | 90-100± 10 | 450-500 | 97.0 ± 7.7 | **C** | 97 ± 3 | 485 | **C** |
| 3_2_P_10_ | **SDX**/PYR:**500**/25 | 85-95 ± 5 | 425-475 | 80-90 ± 0 | 400-450 | 87.5 ± 2.9 | **BLC** | 81 ± 2 | 405 | **NC** |
| 3_2_P_2_ | **SDX**/PYR:**500**/25 | 82-92 ± 4 | 410-460 | 82-92± 4 | 410-460 | 87.5 ± 4.6 | **BLC** | 85 ± 2 | 425 | **NC** |
| 3_3_P_10_ | **SDX**/PYR:**500**/25 | 70-80 ± 5 | 350-400 | 69-79 ± 4 | 345-395 | 74.5 ± 6.0 | **NC** | 82 ± 2 | 410 | **NC** |
| 3_5_P_2_ | **SDX**/PYR:**500**/25 | 50-60 ± 0 | 250-300 | 50-60 ± 0 | 250-300 | 55.0 ± 0.0 | **NC** | 53 ± 3 | 265 | **NC** |
| 3_7_P_15_ | **SDX**/PYR:**500**/25 | 70-80 ± 5 | 350-400 | 75-85 ± 5 | 375-425 | 76.3 ± 6.6 | **NC** | 71 ± 2 | 355 | **NC** |
| 3_8_P_15_ | **SDX**/PYR:**500**/25 | 85-95± 10 | 425-475 | 85-95 ± 5 | 425-475 | 90.0 ± 8.3 | **C** | 91.7 ± 0.2 | 459 | **C** |
| 4_1_P_2_ | **SDX**/PYR:**500**/25 | 85-95 ± 5 | 425-475 | 80-90 ± 5 | 400-450 | 87.5 ± 5.7 | **BLC** | 89 ± 3 | 445 | **BLC** |
| 4_2_P_2_ | **SDX**/PYR:**500**/25 | 55-65 ± 5 | 275-325 | 52-62 ± 4 | 260-310 | 58.5 ± 7.7 | **NC** | 47 ± 2 | 235 | **NC** |
| 4_4_P_2_ | **SDX**/PYR:**500**/25 | 72-82 ± 4 | 360-410 | 75-85 ± 10 | 375-425 | 78.5 ± 8.9 | **NC** | 85.17 ± 0.06 | 426 | **NC** |
| 4_5_P_2_ | **SDX**/PYR:**500**/25 | 82-92 ± 4 | 410-460 | 85-95 ± 5 | 425-475 | 88.5 ± 5.1 | **BLC** | 85.1 ± 0.2 | 426 | **NC** |
| 4_8_P_5_ | **SDX**/PYR:**500**/25 | 87-97 ± 5 | 435-485 | 90-100± 10 | 450-500 | 93.5 ± 8.0 | **C** | 85 ± 2 | 425 | **NC** |
| *2_4_Y_13_ | ATS/**SDX**/PYR:100/**500**/25 | 79-89 ± 4 | 395-445 | 73-83 ± 5 | 365-415 | 81.0 ± 5.6 | **NC** | 72 ± 2 | 360 | **NC** |
| *4Y_13_ | ATS/**SDX**/PYR:100/**500**/25 | 77-87 ± 5 | 385-435 | 77-87 ± 5 | 385-435 | 82.0 ± 6.1 | **NC** | 89 ± 2 | 445 | **BLC** |
| *3_4_Y_13_ | ATS/**SDX**/PYR:100/**500**/25 | 65-75 ± 5 | 325-375 | 63-73 ± 5 | 315-365 | 69.0 ± 7.2 | **NC** | 74 ± 4 | 370 | **NC** |
| *3_2_Y_13_ | ATS/**SDX**/PYR:100/**500**/25 | 64-74 ± 5 | 320-370 | 63-73 ± 5 | 315-365 | 68.5 ± 7.3 | **NC** | 68 ± 5 | 340 | **NC** |
| 4_4_Y_12_ | ATS/**SM**/PYR:100/**250**/12.5 | - | - | - | - | - | - | 90 ± 3 | 225 | **C** |
| 4_2_Y_12_ | ATS/**SM**/PYR:200/**500**/25 | - | - | - | - | - | - | 88.6 ± 0.2 | 443 | **BLC** |
| 4_3_Y_12_ | ATS/**SM**/PYR:100/**250**/12.5 | - | - | - | - | - | - | 87 ± 2 | 218 | **NC** |
| 4_1_Y_12_ | ATS/**SM**/PYR:200/**500**/25 | - | - | - | - | - | - | 87.9 ± 0.5 | 440 | **BLC** |
| 3_1_Y_12_ | ATS/**SM**/PYR:100/**250**/12.5 | - | - | - | - | - | - | 86.6 ± 0.2 | 217 | **NC** |
| 1_5_Z_1_ | DHA/**SDX**/PYR:60/**500**/25 | 84-94 ± 5 | 420-470 | 88-98 ± 4 | 440-490 | 91.0 ± 4.9 | **C** | 85.694 ± 0 | 428 | **NC** |
| 1_3_Z_1_ | DHA/**SDX**/PYR:60/**500**/25 | 84-94± 5 | 420-470 | 82-92 ± 4 | 410-460 | 88.0 ± 5.1 | **BLC** | 86 ± 2 | 430 | **NC** |
| 1_2_Z_1_ | DHA/**SDX**/PYR:60/**500**/25 | 72-82 ± 4 | 360-410 | 70-80 ± 10 | 350-400 | 76.0 ± 9.2 | **NC** | 82.7 ± 0.9 | 414 | **NC** |
| 1_4_Z_1_ | DHA/**SDX**/PYR:60/**500**/25 | 65-75± 10 | 325-375 | 70-80 ± 5 | 350-400 | 72.5 ± 10.3 | **NC** | 79 ± 2 | 395 | **NC** |
| 2_1_Z_1_ | DHA/**SDX**/PYR:60/**500**/25 | 72-82 ± 4 | 360-410 | 78-88 ± 5 | 390-440 | 80.0 ± 5.6 | **NC** | 77 ± 2 | 385 | **NC** |
| 2_3_Z_1_ | DHA/**SDX**/PYR:60/**500**/25 | 90-100± 5 | 450-500 | 90-100 ± 5 | 450-500 | 95.0 ± 5.3 | **C** | 90 ± 2 | 450 | **C** |
| 2_4_Z_1_ | DHA/**SDX**/PYR:60/**500**/25 | 70-80± 10 | 350-400 | 75-85 ± 10 | 375-425 | 77.5 ± 12.9 | **NC** | 80 ± 2 | 400 | **NC** |
| 2_5_Z_1_ | DHA/**SDX**/PYR:60/**500**/25 | 79-89± 4 | 395-445 | 75-85 ± 5 | 375-425 | 82.0 ± 5.5 | **NC** | 86.7 ± 0.6 | 434 | **NC** |
| 3_4_Z_1_ | DHA/**SDX**/PYR:60/**500**/25 | 80-90 ± 5 | 400-450 | 75-85 ± 5 | 375-425 | 82.5 ± 6.1 | **NC** | 85.4 ± 0.2 | 427 | **NC** |
| 3_5_Z_1_ | DHA/**SDX**/PYR:60/**500**/25 | 87-97 ± 5 | 435-485 | 80-90 ± 0 | 400-450 | 88.5 ± 2.8 | **BLC** | 84.9 ± 0.1 | 425 | **NC** |
| 4_5_Z_1_ | DHA/**SDX**/PYR:60/**500**/25 | 74-84 ± 5 | 370-420 | 75-85 ± 5 | 375-425 | 79.5 ± 6.3 | **NC** | 87.5 ± 0.8 | 438 | **NC** |
| 4_6_Z_1_ | DHA/**SDX**/PYR:60/**500**/25 | 74-84 ± 5 | 370-420 | 80-90 ± 5 | 400-450 | 82.0 ± 6.1 | **NC** | 75.2 ± 0.1 | 376 | **NC** |

Summary – 66.67% (26/39) gave the same conclusion for both assays

Table VI: Percentage (%) and Mass (mg) quantities of results of pyrimethamine active pharmaceutical ingredient (API) by TLC and HPLC methods and their comparisons with the manufacturer’s claim and pharmacopoeial requirements. Pyrimethamine tablets must contain at least 90.0% and at most 110.0% of the labelled amount of Pyrimethamine on the pack.

| **Code** | **Manufacturer’s**  **Label Claim (mg)** | **Semi-quantitative TLC estimation of composition in % and mg quantities of dosage forms of pyrimethamine compared to the manufacturer’s label claim n = 6 for each solvent system, total n = 12)** | | | | | **Remarks based on TLC results** | **HPLC determination of composition of pyrimethamine dosage forms in % and mg quantities**  **(n = 6)** | | **Remarks based on HPLC results** |
| --- | --- | --- | --- | --- | --- | --- | --- | --- | --- | --- |
|  |  | **Solvent system 1**  Ethyl Acetate/methanol/ammonia  85:10:5 | | **Solvent system 2**  Ethyl acetate/acetic acid/water  60:20:20 | | **% Average ± rsd** |  |  |  |  |
|  |  | % range ± rsd | Quantity(mg) | % range ± rsd | Quantity(mg) |  |  | % ± rsd | Quantity(mg) |  |
| 1_1_P_10_ | SDX/**PYR**:500/**25** | 80-90 ± 0 | 20-23 | 90-100 ± 0 | 23-25 | 90.0 ± 0.0 | **C** | 92±0 | 23 | **C** |
| 1_1_P_2_ | SDX/**PYR**:500/**25** | 105-115± 10 | 26-29 | 110-120 ± 5 | 28-30 | 112.5 ± 6.7 | **BLC** | 114±0 | 29 | **NC** |
| 1_2_P_2_ | SDX/**PYR**:500/**25** | 69-79 ± 4 | 17-20 | 69-79± 4 | 17-20 | 74.0 ± 5.4 | **NC** | 76±0 | 19 | **NC** |
| 1_4_P_10_ | SDX/**PYR**:500/**25** | 92-102 ± 4 | 23-26 | 94-104 ± 5 | 24-26 | 98.0 ± 4.6 | **C** | 110±0 | 28 | **C** |
| 1_4_P_2_ | SDX/**PYR**:500/**25** | 87-97 ± 5 | 22-24 | 89-99 ± 5 | 22-25 | 93.0 ± 5.4 | **C** | 93±0 | 23 | **C** |
| 1_6_P_10_ | SDX/**PYR**:500/**25** | 87-97 ± 5 | 22-24 | 92-102 ± 4 | 23-26 | 94.5 ± 4.8 | **C** | 92±0 | 23 | **C** |
| 2_1_P_15_ | SDX/**PYR**:500/**25** | 85-95 ± 5 | 21-24 | 85-95 ± 5 | 21-24 | 90.0 ± 5.6 | **C** | 93±0 | 23 | **C** |
| 2_1_P_2_ | SDX/**PYR**:500/**25** | 87-97 ± 5 | 22-24 | 87-97 ± 5 | 22-24 | 92.0 ± 5.4 | **C** | 89±0 | 22 | **BLC** |
| 2_2_P_2_ | SDX/**PYR**:500/**25** | 60-70 ± 10 | 15-18 | 57-67 ± 5 | 14-17 | 63.5 ± 11.8 | **NC** | 64±0 | 16 | **NC** |
| 2_3_P_2_ | SDX/**PYR**:500/**25** | 107-117± 5 | 27-29 | 107-117± 5 | 27-29 | 112.0 ± 4.5 | **BLC** | 122±0 | 31 | **NC** |
| 3_1_P_10_ | SDX/**PYR**:500/**25** | 80-90 ± 10 | 20-23 | 85-95 ± 5 | 21-24 | 87.5 ± 8.6 | **BLC** | 83±0 | 21 | **NC** |
| 3_1_P_2_ | SDX/**PYR**:500/**25** | 92-102 ± 4 | 23-26 | 94-104± 5 | 24-26 | 98.0 ± 4.6 | **C** | 97±0 | 24 | **C** |
| 3_2_P_10_ | SDX/**PYR**:500/**25** | 85-95 ± 5 | 21-24 | 84-94 ± 4 | 21-24 | 89.5 ± 5.0 | **BLC** | 94±0 | 24 | **C** |
| 3_2_P_2_ | SDX/**PYR**:500/**25** | 79-89 ± 4 | 20-22 | 80-90 ± 5 | 20-23 | 84.5 ± 5.3 | **NC** | 96±0 | 24 | **C** |
| 3_3_P_10_ | SDX/**PYR**:500/**25** | 84-94 ± 5 | 21-24 | 82-92± 4 | 21-23 | 88.0 ± 5.1 | **BLC** | 87±0 | 22 | **NC** |
| 3_5_P_2_ | SDX/**PYR**:500/**25** | 40-50 ± 0 | 10-13 | 40-50 ± 0 | 10-13 | 45.0 ± 0.0 | **NC** | 48±0 | 12 | **NC** |
| 3_7_P_15_ | SDX/**PYR**:500/**25** | 80-90 ± 5 | 20-23 | 70-80 ± 0 | 18-20 | 80.0 ± 3.1 | **NC** | 78±0 | 20 | **NC** |
| 3_8_P_15_ | SDX/**PYR**:500/**25** | 99-109 ± 4 | 25-27 | 95-105 ± 5 | 24-26 | 102.0 ± 4.4 | **C** | 103±0 | 26 | **C** |
| 4_1_P_2_ | SDX/**PYR**:500/**25** | 110-120 ± 5 | 28-30 | 110-120 ± 5 | 28-30 | 115.0 ± 4.3 | **BLC** | 113±0 | 28 | **NC** |
| 4_2_P_2_ | SDX/**PYR**:500/**25** | 60-70 ± 5 | 15-18 | 50-60 ± 0 | 13-15 | 60.0 ± 4.2 | **NC** | 51±0 | 13 | **NC** |
| 4_4_P_2_ | SDX/**PYR**:500/**25** | 80-90 ± 15 | 20-23 | 75-85 ± 10 | 19-21 | 82.5 ± 14.6 | **NC** | 102±0 | 26 | **C** |
| 4_5_P_2_ | SDX/**PYR**:500/**25** | 95-105 ± 5 | 24-26 | 99-109 ± 5 | 25-27 | 102.0 ± 4.9 | **C** | 108±0 | 27 | **C** |
| 4_8_P_5_ | SDX/**PYR**:500/**25** | 104-114± 5 | 26-29 | 102-112± 4 | 26-28 | 108.0 ± 4.2 | **C** | 110±0 | 28 | **C** |
| *2_4_Y_13_ | ATS/SDX/**PYR**:100/500/**25** | 85-95 ± 5 | 21-24 | 90-100 ± 0 | 23-25 | 92.5 ± 2.7 | **C** | 91±0 | 23 | **C** |
| *4Y_13_ | ATS/SDX/**PYR**:100/500/**25** | 80-90 ± 5 | 20-23 | 85-95 ± 5 | 21-24 | 87.5 ± 5.7 | **BLC** | 98±0 | 25 | **C** |
| *3_4_Y_13_ | ATS/SDX/**PYR**:100/500/**25** | 60-70 ± 0 | 15-18 | 60-70 ± 0 | 15-18 | 65.0 ± 0.0 | **NC** | 70±0 | 18 | **NC** |
| *3_2_Y_13_ | ATS/SDX/**PYR**:100/500/**25** | 75-85 ± 5 | 19-21 | 72-82 ± 4 | 18-21 | 78.5 ± 5.7 | **NC** | 78±0 | 20 | **NC** |
| 1_5_Z_1_ | DHA/SDX/**PYR**:60/500/**25** | 90-100 ± 0 | 23-25 | 87-97 ± 5 | 22-24 | 93.5 ± 2.7 | **C** | 94±0 | 24 | **C** |
| 1_3_Z_1_ | DHA/SDX/**PYR**:60/500/**25** | 87-97 ± 5 | 22-24 | 88-98 ± 4 | 22-25 | 92.5 ± 4.9 | **C** | 91±0 | 23 | **C** |
| 1_2_Z_1_ | DHA/SDX/**PYR**:60/500/**25** | 90-100 ± 5 | 23-25 | 88-98 ± 4 | 22-25 | 94.0 ± 4.8 | **C** | 98±0 | 25 | **C** |
| 1_4_Z_1_ | DHA/SDX/**PYR**:60/500/**25** | 95-105 ± 5 | 24-26 | 95-105 ± 5 | 24-26 | 97.5 ± 5.1 | **C** | 106±0 | 27 | **C** |
| 2_1_Z_1_ | DHA/SDX/**PYR**:60/500/**25** | 89-99 ± 4 | 22-25 | 88-98 ± 5 | 22-25 | 93.5 ± 4.8 | **C** | 105±0 | 26 | **C** |
| 2_3_Z_1_ | DHA/SDX/**PYR**:60/500/**25** | 95-105 ± 5 | 24-26 | 90-100 ± 0 | 23-25 | 97.5 ± 2.6 | **C** | 100±0 | 25 | **C** |
| 2_4_Z_1_ | DHA/SDX/**PYR**:60/500/**25** | 95-105 ± 5 | 24-26 | 90-100 ± 10 | 23-25 | 97.5 ± 7.7 | **C** | 95±0 | 24 | **C** |
| 2_5_Z_1_ | DHA/SDX/**PYR**:60/500/**25** | 84-94 ± 5 | 21-24 | 90-100 ± 5 | 23-25 | 92.0 ± 5.4 | **C** | 90±0 | 23 | **C** |
| 3_4_Z_1_ | DHA/SDX/**PYR**:60/500/**25** | 90-100 ± 0 | 23-25 | 92-102 ± 4 | 23-26 | 96.0 ± 2.1 | **C** | 98±0 | 25 | **C** |
| 3_5_Z_1_ | DHA/SDX/**PYR**:60/500/**25** | 104-114± 5 | 26-29 | 100-110 ± 0 | 25-28 | 107.0 ± 2.3 | **C** | 98±0 | 25 | **C** |
| 4_5_Z_1_ | DHA/SDX/**PYR**:60/500/**25** | 97-107 ± 5 | 24-27 | 100-110 ± 0 | 25-28 | 103.5 ± 2.4 | **C** | 103±0. | 26 | **C** |
| 4_6_Z_1_ | DHA/SDX/**PYR**:60/500/**25** | 94-104 ± 5 | 24-26 | 94-104 ± 5 | 24-26 | 99.0 ± 5.1 | **C** | 99±0 | 25 | **C** |
| 4_4_Y_12_ | ATS/SM/**PYR**:100/250/**12.5** | 104-114± 5 | 13-14 | 107-117± 5 | 13-15 | 110.5 ± 4.5 | **BLC** | 119±0 | 15 | **NC** |
| 4_2_Y_12_ | ATS/SM/**PYR**:200/500/**25** | 95-105 ± 5 | 24-26 | 107-117± 5 | 27-29 | 106.0 ± 4.7 | **C** | 116±0 | 29 | **NC** |
| 4_3_Y_12_ | ATS/SM/**PYR**:100/250/**12.5** | 115-125 ± 5 | 14-16 | 105-115 ± 10 | 13-14 | 115.0 ± 6.5 | **BLC** | 113±0 | 14 | **NC** |
| 4_1_Y_12_ | ATS/SM/**PYR**:200/500/**25** | 90-100 ± 5 | 23-25 | 85-95 ± 5 | 21-24 | 92.5 ± 5.4 | **C** | 101.73±0 | 25 | **C** |
| 3_1_Y_12_ | ATS/SM/**PYR**:100/250/**12.5** | 99-109 ± 4 | 12-14 | 97-107 ± 5 | 12-13 | 103.0 ± 4.4 | **C** | 110±0 | 14 | **C** |

Summary – 70.45% (31/44) samples gave the same conclusion from both assays

Table VII: Percentage (%) and Mass (mg) quantities of results of quinine active pharmaceutical ingredient (API) by TLC and HPLC methods and their comparisons with the manufacturer’s claim and pharmacopoeial requirements. Quinine tablets must contain at least 90.0% and at most 110.0% of the labelled amount of Quinine on the pack

| **Code** | **Manufacturer’s**  **Label Claim (mg)** | **Semi-quantitative TLC estimation of composition in % and mg quantities of dosage forms of quinine compared to the manufacturer’s label claim n = 6 for each solvent system, total n = 12)** | | | | | **Remarks based on TLC results** | **HPLC determination of composition of quinine dosage forms in % and mg quantities**  **(n = 6)** | | **Remarks based on HPLC results** |
| --- | --- | --- | --- | --- | --- | --- | --- | --- | --- | --- |
|  |  | **Solvent system 1**  Methanol: ammonia  100:1.5 | | **Solvent system 2**  Ethyl acetate: acetic acid: water  60:20:20 | | **% Average ± rsd** |  |  |  |  |
|  |  | % range ±sd | Quantity (mg) | % range ±sd | Quantity (mg) |  |  | % ±rsd | Quantity (mg) |  |
| 1_1_V_5_ | **QUN:50mg/5ml** | 102-112 ± 4 | 51-56 | 102-112 ± 4 | 51-56 | 107.0 ± 3.7 | **C** | 109.7 ± 0.3 | 55 | **C** |
| 1_2_V_5_ | **QUN:50mg/5ml** | 110-120 ± 5 | 55-60 | 103-113 ± 10 | 52-57 | 111.0 ± 6.8 | **BLC** | 120 ± 1 | 60 | **NC** |
| 1_3_V_5_ | **QUN:50mg/5ml** | 104-114 ± 5 | 52-57 | 104-114 ± 5 | 52-57 | 109.0 ± 4.6 | **C** | 112 ± 1 | 56 | **BLC** |
| 4V_5_ | **QUN:50mg/5ml** | 107-117± 5 | 54-59 | 107-117± 5 | 54-59 | 112.0 ± 4.5 | **BLC** | 112 ± 2 | 56 | **BLC** |
| 4_1_R_8_ | **QUN:100mg/5ml** | 130-140 ± 10 | 130-140 | 135-145 ± 5 | 135-145 | 137.5 ± 5.5 | **NC** | 291 ± 1 | 291 | **NC** |
| 4_2_R_4_ | **QUN:100mg/5ml** | 125-135 ± 5 | 125-135 | 125-135 ± 5 | 125-135 | 130.0 ± 3.8 | **NC** | 151 ± 1 | 151 | **NC** |
| 4_3_R_4_ | **QUN:100mg/5ml** | 117-127± 5 | 117-127 | 120-130 ± 0 | 120-130 | 123.0 ± 2.0 | **NC** | 156 ± 2 | 156 | **NC** |
| *4_1_Q_6_ | **QUN:50mg/5ml** | - | - | - | - |  |  | 102 ± 4 | 51 | **C** |
| *4_2_Q_6_ | **QUN:50mg/5ml** | - | - | - | - |  |  | 56 ± 7 | 28 | **NC** |
| *4_3_Q_6_ | **QUN:50mg/5ml** | - | - | - | - |  |  | 97 ± 4 | 49 | **C** |
| *3_1_Q_6_ | **QUN:150mg** | - | - | - | - |  |  | 110.4 ± 0.1 | 166 | **C** |
| *3_2_Q_6_ | **QUN:150mg** | - | - | - | - |  |  | 110.5 ± 0.1 | 166 | **C** |
| *3_3_Q_6_ | **QUN:150mg** | - | - | - | - |  |  | 122.1 ± 0.8 | 183 | **NC** |

* samples could not be analysed by SQ-TLC due to technical reasons

Summary 71.43% (5/7) samples gave the same conclusion for both assays
