# Supplementary material for: Further Validation of a Rapid Screening Semiquantitative Thin-Layer Chromatographic Method for Marketed Antimalarial Medicines for Adoption in Malawi
Source: J Anal Methods Chem. 2018 May 2;2018:2130390. doi: 10.1155/2018/2130390 (PMC5954873; doi:10.1155/2018/2130390)
Supplement: Supplementary 2. — HPLC and SQ-TLC results for antimalarial samples. [file 2130390.f2.docx]

**SUPPLEMENTARY FILE 2**

**HPLC AND SQ-TLC RESULTS FOR ANTIMALARIAL SAMPLES**

Table Ia: Quality evaluation of antimalarial samples containing **Artesunate**, **Sulphadoxine** and **Pyrimethamine** active pharmaceutical ingredients (APIs) based on the quality of the individual API components

| **Code** | **Manufacturer’s**  **Label Claim (mg)** | SQ-TLC results | | | |  | HPLC results | | | |
| --- | --- | --- | --- | --- | --- | --- | --- | --- | --- | --- |
|  |  | Quality of individual API components | | | Quality of sample as a whole |  | Quality of individual API components | | | Quality of sample as a whole |
|  |  | **ATS** | **SDX** | **PYR** |  |  | **ATS** | **SDX** | **PYR** |  |
| *2_4_Y_13_ | ATS/SDX/PYR:100/500/25 | BLC | NC | C | **NC** |  | C | NC | C | **NC** |
| *4Y_13_ | ATS/SDX/PYR:100/500/25 | C | NC | BLC | **NC** |  | C | BLC | C | **BLC** |
| *3_4_Y_13_ | ATS/SDX/PYR:100/500/25 | C | NC | NC | **NC** |  | C | NC | NC | **NC** |
| *3_2_Y_13_ | ATS/SDX/PYR:100/500/25 | C | NC | NC | **NC** |  | C | NC | NC | **NC** |

**L62.5)antial portion (72ant – 6** For the combination therapies, a sample was considered compliant (C) if all component APIs were compliant; noncompliant (NC) if one or more of the API components was NC and borderline compliant (BLC) if all components were BLC or there was one or more BLC component among C components.

Table Ib: Summary of results for quality evaluation of antimalarial samples containing **Artesunate**, **Sulphadoxine** and **Pyrimethamine** APIs by TLC and HPLC

| **ATS/SDX/PYR (4 samples)** | **C** | **BLC** | **NC** |
| --- | --- | --- | --- |
| **SQ-TLC** | 0 | 0 | 4 (100%) |
| **HPLC** | 0 | 1 (25%) | 3 (75%) |

75% (3/4) samples gave same results for both assays

Table IIa: Quality evaluation of the antimalarial samples containing **Artesunate**, **Sulphamethoxypyridazine** and **Pyrimethamine** active pharmaceutical ingredients (APIs) based on the quality of the individual API components

| **Code** | **Manufacturer’s**  **Label Claim (mg)** | SQ-TLC results | | | |  | HPLC results | | | |
| --- | --- | --- | --- | --- | --- | --- | --- | --- | --- | --- |
|  |  | Quality of individual API components | | | Quality of sample as a whole |  | Quality of individual API components | | | Quality of sample as a whole |
|  |  | **ATS** | **SM** | **PYR** |  |  | **ATS** | **SM** | **PYR** |  |
| 4_4_Y_12_ | ATS/SM/PYR:100/250/12 | C | - | BLC | **BLC** |  | C | C | NC | **NC** |
| 4_2_Y_12_ | ATS/SM/PYR:200/500/25 | C | - | C | **C** |  | C | BLC | NC | **NC** |
| 4_3_Y_12_ | ATS/SM/PYR:100/250/12.5 | NC | - | BLC | **NC** |  | NC | NC | NC | **NC** |
| 4_1_Y_12_ | ATS/SM/PYR:200/500/25 | C | - | C | **C** |  | NC | BLC | C | **NC** |
| 3_1_Y_12_ | ATS/SM/PYR:200/500/25 | NC | - | C | **NC** |  | BLC | NC | C | **NC** |

**L62.5)antial portion (72ant – 6**SM = sulphamethoxypyridazine. On TLC, it gave a spot with the same retention time as sulphadoxine, but it failed to stain well with I_2_/KI for SQ-TLC analysis. However, HPLC analysis was successful.

Table IIb: Summary of results for antimalarial samples containing **Artesunate**, **Sulphamethoxypyridazine** and **Pyrimethamine** APIs by TLC and HPLC

| **ATS/SM/PYR (5 samples)** | **C** | **BLC** | **NC** |
| --- | --- | --- | --- |
| ***SQ-TLC** | 2 (40%) | 1 (20%) | 2 (40%) |
| **HPLC** | 0 | 0 | 5 (100%) |

*these results are based on only 2 APIs instead of 3

40% (2/5) samples gave same results for both assays

Table IIIa: Quality evaluation of the antimalarial samples containing **Artemether** and **Lumefantrine** active pharmaceutical ingredients (APIs) based on the quality of the individual API components

| **Code** | **Manufacturer’s**  **Label Claim (mg)** | SQ-TLC results | | |  | HPLC results | | |
| --- | --- | --- | --- | --- | --- | --- | --- | --- |
|  |  | Quality of individual API components | | Quality of sample as a whole |  | Quality of individual API components | | Quality of sample as a whole |
|  |  | **ATM** | **LUM** |  |  | **ATM** | **LUM** |  |
| 2X_15_ | ATM/LUM:20/120 | NC | C | **NC** |  | NC | C | **NC** |
| 4X_20_ | ATM/LUM:20/120 | C | NC | **NC** |  | C | NC | **NC** |
| 1_1_X_1_ | ATM/LUM:20/120 | NC | C | **NC** |  | NC | NC | **NC** |
| 1_1_X_11_ | ATM/LUM:180/1080 | C | NC | **NC** |  | C | NC | **NC** |
| 1_1_X_14_ | ATM/LUM:20/120 | NC | NC | **NC** |  | NC | NC | **NC** |
| 1_1_X_17_ | ATM/LUM:80/480 | NC | C | **NC** |  | NC | C | **NC** |
| 1_1_X_18_ | ATM/LUM:40/240 | NC | C | **NC** |  | NC | C | **NC** |
| 1_1_X_20_ | ATM/LUM:20/120 | NC | BLC | **NC** |  | NC | NC | **NC** |
| 1_2_X_1_ | ATM/LUM:20/120 | NC | C | **NC** |  | NC | NC | **NC** |
| 1_2_X_11_ | ATM/LUM:180/1080 | C | NC | **NC** |  | C | NC | **NC** |
| 1_2_X_14_ | ATM/LUM:20/120 | NC | C | **NC** |  | NC | C | **NC** |
| 1_3_X_1_ | ATM/LUM:40/240 | C | NC | **NC** |  | C | NC | **NC** |
| 1_3_X_11_ | ATM/LUM:80/480 | NC | NC | **NC** |  | NC | NC | **NC** |
| 1_4_X_1_ | ATM/LUM:40/240 | NC | C | **NC** |  | NC | NC | **NC** |
| 1_4_X_11_ | ATM/LUM:40/240 | NC | C | **NC** |  | NC | NC | **NC** |
| 1_5_X_1_ | ATM/LUM:80/480 | NC | C | **NC** |  | NC | BLC | **NC** |
| 1_5_X_11_ | ATM/LUM:20/120 | NC | C | **NC** |  | NC | C | **NC** |
| 1_6_X_1_ | ATM/LUM:40/240 | NC | NC | **NC** |  | NC | NC | **NC** |
| 1_7_X_1_ | ATM/LUM:80/480 | BLC | NC | **NC** |  | NC | NC | **NC** |
| 1_8_X_1_ | ATM/LUM:20/120 | NC | NC | **NC** |  | NC | NC | **NC** |
| 1_9_X_1_ | ATM/LUM:80/480 | C | BLC | **BLC** |  | C | NC | **NC** |
| 2_1_X_1_ | ATM/LUM:20/120 | C | BLC | **BLC** |  | C | NC | **NC** |
| 2_2_X_1_ | ATM/LUM:80/480 | NC | NC | **NC** |  | NC | NC | **NC** |
| 2_3_X_1_ | ATM/LUM:20/120 | NC | C | **NC** |  | NC | C | **NC** |
| 2_4_X_14_ | ATM/LUM:20/120 | NC | NC | **NC** |  | NC | NC | **NC** |
| 2_5_X_11_ | ATM/LUM:80/480 | BLC | C | **BLC** |  | NC | NC | **NC** |
| 3_1_X_1_ | ATM/LUM:20/120 | BLC | NC | **NC** |  | C | NC | **NC** |
| 3_2_X_1_ | ATM/LUM:20/120 | C | NC | **NC** |  | C | NC | **NC** |
| 3_1_X_11_ | ATM/LUM:80/480 | NC | C | **NC** |  | NC | NC | **NC** |
| 3_3_X_1_ | ATM/LUM:40/240 | NC | C | **NC** |  | NC | C | **NC** |
| 3_4_X_1_ | ATM/LUM:80/480 | C | C | **C** |  | C | BLC | **BLC** |
| 3_6_X_1_ | ATM/LUM:80/480 | C | C | **C** |  | C | C | **C** |
| 4_1_X_11_ | ATM/LUM:20/120 | C | NC | **NC** |  | C | NC | **NC** |
| 4_2_X_11_ | ATM/LUM:20/120 | NC | C | **NC** |  | NC | BLC | **NC** |
| 4_3_X_1_ | ATM/LUM:40/240 | NC | NC | **NC** |  | NC | NC | **NC** |
| 4_4_X_1_ | ATM/LUM:80/480 | NC | C | **NC** |  | NC | C | **NC** |
| 4_5_X_12_ | ATM/LUM:180/1080 | C | NC | **NC** |  | C | NC | **NC** |
| 1_10_X_1_ | ATM/LUM:80/480 | NC | NC | **NC** |  | NC | NC | **NC** |
| 1_6_X_11_ | ATM/LUM:180/1080 | C | BLC | **BLC** |  | C | NC | **NC** |
| 1_12_X_1_ | ATM/LUM:80/480 | NC | C | **NC** |  | C | C | **C** |
| 1_13_X_1_ | ATM/LUM:80/480 | NC | C | **NC** |  | NC | C | **NC** |

Table IIIb: Summary of results for antimalarial samples containing **Artemether** and **Lumefantrine** APIs by TLC and HPLC

| **ATM/LUM (41 samples)** | **C** | **BLC** | **NC** |
| --- | --- | --- | --- |
| **SQ-TLC** | 2 (4.88%) | 4 (9.76%) | 35 (85.36%) |
| **HPLC** | 2 (4.88) | 1 (2.44%) | 38 (92.68%) |

85.36% (35/41) samples gave same results for both assays

Table IVa: Quality evaluation of the antimalarial samples containing **Dihydroartemisinin,** **Sulphadoxine** and **Pyrimethamine** active pharmaceutical ingredients (APIs) based on the quality of the individual API components

| **Code** | **Manufacturer’s**  **Label Claim (mg)** | SQ-TLC results | | | |  | HPLC results | | | |
| --- | --- | --- | --- | --- | --- | --- | --- | --- | --- | --- |
|  |  | Quality of individual API components | | | Quality of sample as a whole |  | Quality of individual API components | | | Quality of sample as a whole |
|  |  | **DHA** | **SDX** | **PYR** |  |  | **DHA** | **SDX** | **PYR** |  |
| 1_5_Z_1_ | DHA/SDX/PYR:60/500/25 | **NC** | **C** | **C** | **NC** |  | **NC** | **NC** | **C** | **NC** |
| 1_3_Z_1_ | DHA/SDX/PYR:60/500/25 | **NC** | **BLC** | **C** | **NC** |  | **NC** | **NC** | **C** | **NC** |
| 1_2_Z_1_ | DHA/SDX/PYR:60/500/25 | **NC** | **NC** | **C** | **NC** |  | **NC** | **NC** | **C** | **NC** |
| 1_4_Z_1_ | DHA/SDX/PYR:60/500/25 | **NC** | **NC** | **C** | **NC** |  | **NC** | **NC** | **C** | **NC** |
| 2_1_Z_1_ | DHA/SDX/PYR:60/500/25 | **NC** | **NC** | **C** | **NC** |  | **NC** | **NC** | **C** | **NC** |
| 2_3_Z_1_ | DHA/SDX/PYR:60/500/25 | **NC** | **C** | **C** | **NC** |  | **N C** | **C** | **C** | **NC** |
| 2_4_Z_1_ | DHA/SDX/PYR:60/500/25 | **NC** | **NC** | **C** | **NC** |  | **NC** | **NC** | **C** | **NC** |
| 2_5_Z_1_ | DHA/SDX/PYR:60/500/25 | **NC** | **NC** | **C** | **NC** |  | **NC** | **NC** | **C** | **NC** |
| 3_4_Z_1_ | DHA/SDX/PYR:60/500/25 | **NC** | **NC** | **C** | **NC** |  | **NC** | **NC** | **C** | **NC** |
| 3_5_Z_1_ | DHA/SDX/PYR:60/500/25 | **NC** | **BLC** | **C** | **NC** |  | **NC** | **NC** | **C** | **NC** |
| 4_5_Z_1_ | DHA/SDX/PYR:60/500/25 | **NC** | **NC** | **C** | **NC** |  | **NC** | **NC** | **C** | **NC** |
| 4_6_Z_1_ | DHA/SDX/PYR:60/500/25 | **NC** | **NC** | **C** | **NC** |  | **NC** | **NC** | **C** | **NC** |

Table IVb: Summary of results for antimalarial samples containing **Dihydroartemisinin,** **Sulphadoxine** and **Pyrimethamine** APIs by TLC and HPLC

| **DHA/SDX/PYR (12 samples)** | **C** | **BLC** | **NC** |
| --- | --- | --- | --- |
| **SQ-TLC** | 0 | 0 | 12 (100%) |
| **HPLC** | 0 | 0 | 12 (100%) |

100% (12/12) samples gave same results for both assays

Table Va: Quality evaluation of the antimalarial samples containing **Sulphadoxine** and **Pyrimethamine** active pharmaceutical ingredients (APIs) based on the quality of the individual API components

| **Code** | **Manufacturer’s**  **Label Claim (mg)** | SQ-TLC results | | |  | HPLC results | | |
| --- | --- | --- | --- | --- | --- | --- | --- | --- |
|  |  | Quality of individual API components | | Quality of sample as a whole |  | Quality of individual API components | | Quality of sample as a whole |
|  |  | **SDX** | **PYR** |  |  | **SDX** | **PYR** |  |
| 1_1_P_10_ | **SDX**/PYR:**500**/25 | NC | C | **NC** |  | NC | C | **NC** |
| 1_1_P_2_ | **SDX**/PYR:**500**/25 | BLC | BLC | **BLC** |  | NC | NC | **NC** |
| 1_2_P_2_ | **SDX**/PYR:**500**/25 | NC | NC | **NC** |  | NC | NC | **NC** |
| 1_4_P_10_ | **SDX**/PYR:**500**/25 | NC | C | **NC** |  | NC | C | **NC** |
| 1_4_P_2_ | **SDX**/PYR:**500**/25 | NC | C | **NC** |  | NC | C | **NC** |
| 1_6_P_10_ | **SDX**/PYR:**500**/25 | BLC | C | **BLC** |  | NC | C | **NC** |
| 2_1_P_15_ | **SDX**/PYR:**500**/25 | NC | C | **NC** |  | NC | C | **NC** |
| 2_1_P_2_ | **SDX**/PYR:**500**/25 | BLC | C | **BLC** |  | NC | BLC | **NC** |
| 2_2_P_2_ | **SDX**/PYR:**500**/25 | BLC | NC | **NC** |  | NC | NC | **NC** |
| 2_3_P_2_ | **SDX**/PYR:**500**/25 | C | BLC | **BLC** |  | C | NC | **NC** |
| 3_1_P_10_ | **SDX**/PYR:**500**/25 | C | BLC | **BLC** |  | NC | NC | **NC** |
| 3_1_P_2_ | **SDX**/PYR:**500**/25 | C | C | **C** |  | C | C | **C** |
| 3_2_P_10_ | **SDX**/PYR:**500**/25 | BLC | BLC | **BLC** |  | NC | C | **NC** |
| 3_2_P_2_ | **SDX**/PYR:**500**/25 | BLC | NC | **NC** |  | NC | C | **NC** |
| 3_3_P_10_ | **SDX**/PYR:**500**/25 | NC | BLC | **NC** |  | NC | NC | **NC** |
| 3_5_P_2_ | **SDX**/PYR:**500**/25 | NC | NC | **NC** |  | NC | NC | **NC** |
| 3_7_P_15_ | **SDX**/PYR:**500**/25 | NC | NC | **NC** |  | NC | NC | **NC** |
| 3_8_P_15_ | **SDX**/PYR:**500**/25 | C | C | **C** |  | C | C | **C** |
| 4_1_P_2_ | **SDX**/PYR:**500**/25 | BLC | BLC | **BLC** |  | BLC | NC | **NC** |
| 4_2_P_2_ | **SDX**/PYR:**500**/25 | NC | NC | **NC** |  | NC | NC | **NC** |
| 4_4_P_2_ | **SDX**/PYR:**500**/25 | NC | NC | **NC** |  | NC | C | **NC** |
| 4_5_P_2_ | **SDX**/PYR:**500**/25 | BLC | C | **BLC** |  | NC | C | **NC** |
| 4_8_P_5_ | **SDX**/PYR:**500**/25 | C | C | **C** |  | NC | C | **NC** |

Table Vb: Summary of results for antimalarial samples containing **Sulphadoxine** and **Pyrimethamine** APIs by TLC and HPLC

| **SDX/PYR (23 samples)** | **C** | **BLC** | **NC** |
| --- | --- | --- | --- |
| **SQ-TLC** | 3 (13.04%) | 8 (34.78%) | 12 (52.17%) |
| **HPLC** | 2 (8.70%) | 0 | 21 (91.30%) |

53.84% (14/23) samples gave same results for both assays

Table VIa: Quality evaluation of the antimalarial samples containing **Dihydroartemisinin** and **Piperaquine** active pharmaceutical ingredients (APIs) based on the quality of the individual API components

| **Code** | **Manufacturer’s**  **Label Claim (mg)** | SQ-TLC results based on DHA API | HPLC results based on DHA API |
| --- | --- | --- | --- |
| 1_1_Z_3_ | **DHA**/PPQ:**40**/320 | **C** | **C** |
| 1_2_Z_3_ | **DHA**/PPQ:**40**/320 | **BLC** | **NC** |
| 1_4_Z_3_ | **DHA**/PPQ:**40**/320 | **NC** | **NC** |
| 1_6_Z_1_ | **DHA**/PPQ:**40**/320 | **C** | **C** |
| 1_7_Z_1_ | **DHA**/PPQ:**40**/320 | **C** | **NC** |
| 2_6_Z_1_ | **DHA**/PPQ:**40**/320 | **C** | **C** |
| 2_7_Z_1_ | **DHA**/PPQ:**40**/320 | **C** | **BLC** |
| 3_2_Z_3_ | **DHA**/PPQ:**40**/320 | **NC** | **NC** |
| 3_3_Z_3_ | **DHA**/PPQ:**40**/320 | **NC** | **NC** |
| 3_7_Z_1_ | **DHA**/PPQ:**40**/320 | **C** | **C** |
| 4_1_Z_3_ | **DHA**/PPQ:**40**/320 | **NC** | **NC** |
| 4_2_Z_3_ | **DHA**/PPQ:**40**/320 | **C** | **NC** |
| 4_3_Z_3_ | **DHA**/PPQ:**40**/320 | **NC** | **NC** |
| 4_4_Z_3_ | **DHA**/PPQ:**40**/320 | **C** | **BLC** |

The piperaquine component could not be analysed due to the absence of a reference substance. Thus the quality evaluation was based only on dihydroartemisinin API.

Table VIb: Summary of results for antimalarial samples containing **Dihydroartemisinin** and **Piperaquine** APIs by TLC and HPLC

| **DHA**/PPQ **(14 samples)** | **C** | **BLC** | **NC** |
| --- | --- | --- | --- |
| **SQ-TLC** | 8 (57.14%) | 1 (7.14%) | 5 (35.71%) |
| **HPLC** | 4 (28.57%) | 2 (14.29%) | 8 (57.14%) |

64.28% (9/14) samples gave same results for both assays

Table VIIa: Quality evaluation of the antimalarial samples containing **Quinine** API

| **Code** | **Manufacturer’s**  **Label Claim (mg)** | SQ-TLC results | HPLC results |
| --- | --- | --- | --- |
| 1_1_V_5_ | **QUN:50mg/5ml** | **C** | **C** |
| 1_2_V_5_ | **QUN:50mg/5ml** | **BLC** | **NC** |
| 1_3_V_5_ | **QUN:50mg/5ml** | **C** | **BLC** |
| 4V_5_ | **QUN:50mg/5ml** | **BLC** | **BLC** |
| 4_1_R_8_ | **QUN:100mg/5ml** | **NC** | **NC** |
| 4_2_R_4_ | **QUN:100mg/5ml** | **NC** | **NC** |
| 4_3_R_4_ | **QUN:100mg/5ml** | **NC** | **NC** |
| *4_1_Q_6_ | **QUN:50mg/5ml** | **-** | **C** |
| *4_2_Q_6_ | **QUN:50mg/5ml** | **-** | **NC** |
| *4_3_Q_6_ | **QUN:50mg/5ml** | **-** | **C** |
| *3_1_Q_6_ | **QUN:150mg** | **-** | **C** |
| *3_2_Q_6_ | **QUN:150mg** | **-** | **C** |
| *3_3_Q_6_ | **QUN:150mg** | **-** | **NC** |

* These samples could not be analysed by TLC for technical reasons

Table VIIb: Summary of results for antimalarial samples containing **Quinine** API by TLC and HPLC

| **QN (7 out of 13 samples)** | **C** | **BLC** | **NC** |
| --- | --- | --- | --- |
| **SQ-TLC** | 2 (28.57%) | 2 (28.57%) | 3 (42.86%) |
| **HPLC** | 1 (14.28%) | 2 (28.57%) | 4 (57.14%) |

71.43% (5/7) samples gave same results for both assays
